# Loss-of-function mutations of *SCN10A* encoding Na_V_1.8 α subunit of voltage-gated sodium channel in patients with human kidney stone disease

**DOI:** 10.1038/s41598-018-28623-3

**Published:** 2018-07-11

**Authors:** Choochai Nettuwakul, Oranud Praditsap, Nunghathai Sawasdee, Nanyawan Rungroj, Katesirin Ruamyod, Wattana B. Watanapa, Mutita Junking, Sittideth Sangnual, Suchai Sritippayawan, Boonyarit Cheunsuchon, Duangporn Chuawattana, Santi Rojsatapong, Wipada Chaowagul, Sulayman D. Dib-Hajj, Stephen G. Waxman, Pa-thai Yenchitsomanus

**Affiliations:** 1 Division of Molecular Medicine, Research Department, Faculty of Medicine Siriraj Hospital, Mahidol University, Bangkok, 10700 Thailand; 2 Division of Molecular Genetics, Research Department, Faculty of Medicine Siriraj Hospital, Mahidol University, Bangkok, 10700 Thailand; 3 Immunology Graduate Program and Department of Immunology, Faculty of Medicine Siriraj Hospital, Mahidol University, Bangkok, 10700 Thailand; 4 Department of Physiology, Faculty of Medicine Siriraj Hospital, Mahidol University, Bangkok, 10700 Thailand; 5 Division of Nephrology, Department of Medicine, Faculty of Medicine Siriraj Hospital, Mahidol University, Bangkok, 10700 Thailand; 6 Department of Pathology, Faculty of Medicine Siriraj Hospital, Mahidol University, Bangkok, 10700 Thailand; 7 Department of Medicine, Sappasithiprasong Hospital, Ubon Ratchathani, 34000 Thailand; 8 Department of Neurology & The Center for Neuroscience and Regeneration Research, Yale University School of Medicine, New Haven, CT 06516 USA

## Abstract

Human kidney stone disease (KSD) causes significant morbidity and public health burden worldwide. The etiology of KSD is heterogeneous, ranging from monogenic defects to complex interaction between genetic and environmental factors. However, the genetic defects causing KSD in the majority of affected families are still unknown. Here, we report the discovery of mutations of *SCN10A*, encoding Na_V_1.8 α subunit of voltage-gated sodium channel, in families with KSD. The region on chromosome 3 where *SCN10A* locates was initially identified in a large family with KSD by genome-wide linkage analysis and exome sequencing. Two mutations (p.N909K and p.K1809R) in the same allele of *SCN10A* co-segregated with KSD in the affected family. Additional mutation (p.V1149M) of *SCN10A* was identified in another affected family, strongly supporting the causal role of *SCN10A* for KSD. The amino acids at these three positions, N909, K1809, and V1149, are highly conserved in vertebrate evolution, indicating their structural and functional significances. Na_V_1.8 α subunit mRNA and protein were found to express in human kidney tissues. The mutant proteins expressed in cultured cells were unstable and causing reduced current density as analyzed by whole-cell patch-clamp technique. Thus, loss-of-function mutations of *SCN10A* were associated with KSD in the families studied.

## Introduction

Kidney stone disease (KSD) is a common disorder affecting approximately 1–5% of the population worldwide^1^. The causes of KSD are heterogeneous, ranging from monogenic defect to complex interaction between genetic and environmental factors^2^. The majority of kidney stones (~80%) are composed of calcium oxalate and calcium phosphate^3,4^. Studies in families and twins have indicated a genetic predisposition to calcium stones^5,6^. Family-based or case-control studies of individual candidate genes in KSD have shown the association with variations in several genes, including *osteopontin (OPN)*^7,8^, *calcitonin receptor (CTR)*^9^, *vitamin D receptor (VDR)*^10,11^, and *calcium-sensing receptor (CaSR)*^12^. A high-throughput genome-wide association study (GWAS) identified sequence variations in *claudin 14 (CLDN14)*, encoding for the tight junction protein expressed in the kidney, liver, and inner ear, associated with KSD^13^.

Previously, our group studied KSD in the Northeastern (NE) population of Thailand, where the disease is prevalent. We have demonstrated that the relative risk of KSD among first-degree relatives and members of affected families was higher than that of the general population (λ_R_ = 3.18), indicating familial aggregation and the role of genetic factors in the pathogenesis^14^. We then evaluated genetic variations associated with KSD in the patients by genotyping single nucleotide polymorphisms (SNP) distributed within 8 candidate genes, including *TFF1*, *S100A8*, *S100A9*, *S100A12*, *AMBP*, *SPP1*, *UMOD*, and *F2*, encoding stone inhibitor proteins. We found that genetic variation of *F2* was associated with KSD risk in the NE Thai female patients^15^ and later reported the association between *F2* variant (p.T165M) and KSD in the NE Thai female patients^16^. However, the *F2* variant (p.T165M) was likely a modifying, not a disease-causing, variation associated with KSD in this population because both variants (T and M) were observed in the patient and control groups with different frequencies. In the present work, we continued the study to identify disease-causing genes for KSD by genome-wide linkage analysis and exome sequencing in the affected families. Here, we provide the evidence that loss-of-function mutations of sodium voltage-gated channel alpha subunit 10, *SCN10A* (MIM: 604427), encoding the Na_V_1.8 α subunit of voltage-gated sodium channel, associate with KSD in the families studied.

## Results

### Subjects and clinical study

**Figure 1:**
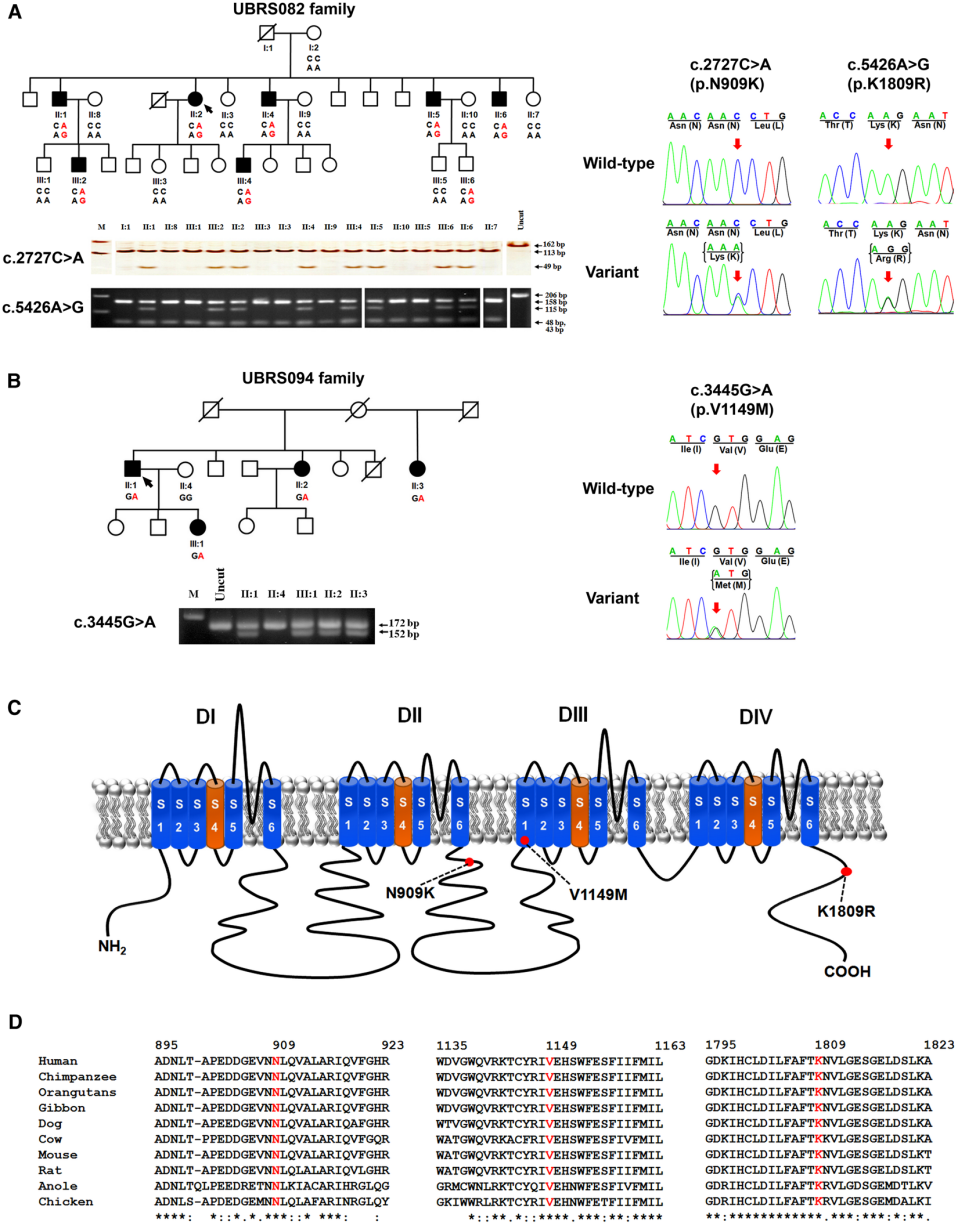
Segregation analysis of KSD and *SCN10A* variations in two affected families, schematic diagram of the Na_V_1.8 α subunit structure, and multiple amino-acid sequence alignment of Na_V_1.8 α subunit. (**A**) Co-segregation of KSD and two *SCN10A* variations [c.2727 C > A (p.N909K), and c.5426 A > G (p.K1809R)] in the UBRS082 family, and sequencing profile of the variations. One member (III:6), aged 15 years, carried the variations without KSD; this may be explained by the late-onset nature (>25 years) of the disease. (**B**) Co-segregation of KSD and *SCN10A* variation [c.3445 G > A (p.V1149M)] in the UBRS094 family, and sequencing profiles of the variations. (**C**) Schematic diagram of the Na_V_1.8 α subunit of voltage-gated sodium channel and distribution of mutations. The red dots indicate the approximate locations of the three identified variations (p.N909K, p.V1149M, and p.K1809R). (**D**) Multiple amino-acid sequence alignments of Na_V_1.8 α subunit from six vertebrate species in three regions where the variations (p.N909K, p.V1149M, and p.K1809R) were identified, which indicate that asparagine (N) at position 909, valine (V) at position 1149, and lysine (K) at position 1809 are highly conserved. Images of the gels cropped from different parts of the same gel, or from different gels were separated by white space. The full-length gels are presented in Supplementary Fig. S8.

A large index family with KSD (UBRS082) was selected from 180 unrelated families for genome-wide linkage analysis and exome sequencing. Seventeen family members were investigated for KSD by kidney-ureter-bladder (KUB) radiography, clinical history, physical examination, blood and urine biochemical analyses (Table 1). The KSD phenotype was first detected in the proband (II-2) and inherited as autosomal dominant model (Fig. 1A) with 7 affected and 10 unaffected members; the father in the first generation (I:1) who was deceased was unknown for diagnosis status. All affected members were new KSD cases diagnosed at different times. No hypercalciuria, hyperoxaluria or hyperphosphaturia was observed in this family. Clinical characteristics and symptoms were summarized in Table 1. All stones present in the affected members were radio opaque, indicating their calcium content. Three affected members (II:1, II:2, and III:2) had surgery long time ago; their stones were unable to be collected for component analysis. Our previous data showed that calcium stones with opaque appearance on KUB were observed in about 88% of stones analyzed in this population^14^.

**Table 1:** Some clinical and laboratory data of the members of the UBRS082 family.

Sample No.	Gender	Age	Age of Onset^*^	KUB result	Site/Side of stone	No. of stone	Treatment	Other Symptoms				Diagnosis
								Dysuria	Hematuria	Pass Stone	Turbid Urine	
II:1	Male	54	15	Negative	Unknown/ Right	N/A	Surgery	Yes	Yes	Yes	Yes	renal stone (surgical scar^1^)
II:2	Female	51	38	Positive	Ureter/ Right	1	Surgery	Yes	No	No	No	ureteric stone (KUB^2^)
II:4	Male	47	30	Positive	Renal/Right	1	No	Yes	No	Yes	Yes	renal stone (KUB)
II:5	Male	39	N/A	Positive	Renal/Left	1	No	No	No	No	No	renal stone (KUB)
II:6	Male	36	13	Positive	Renal/ Both	>5	No	Yes	No	No	No	renal stone (KUB)
III:2	Male	31	21	Positive	Renal/Left	>5	Surgery	Yes	No	No	No	renal stone (KUB)
III:4	Male	27	21	Negative	N/A	N/A	No	Yes	No	Yes	Yes	renal stone (strong clinical history^3^)
I:2	Female	76		Negative			No	No	No	No	No	no stone
II:3	Female	49		Negative			No	No	No	No	No	no stone
II:7	Female	35		Negative			No	No	No	No	No	no stone
II:8	Female	53		Negative			No	No	No	No	No	no stone
II:9	Female	45		Negative			No	Yes	Yes	No	No	no stone
II:10	Female	39		Negative			No	No	No	No	No	no stone
III:1	Male	33		Negative			No	No	No	No	No	no stone
III:3	Female	29		Negative			No	No	No	No	No	no stone
III:5	Male	17		Negative			No	No	No	No	No	no stone
III:6	Male	15		Negative			No	No	No	No	No	no stone

^1^The patient had surgical scar of kidney stone removal.

^2^The patient had positive results of kidney–ureter–bladder (KUB) radiography.

^3^The patient had strong clinical history as justified from the presence of several symptoms associated with kidney stone, especially hematuria and stone passage.

^*^Age at the onset of kidney stone disease.

### Genome-wide linkage analysis and exome sequencing

**Figure 2:**
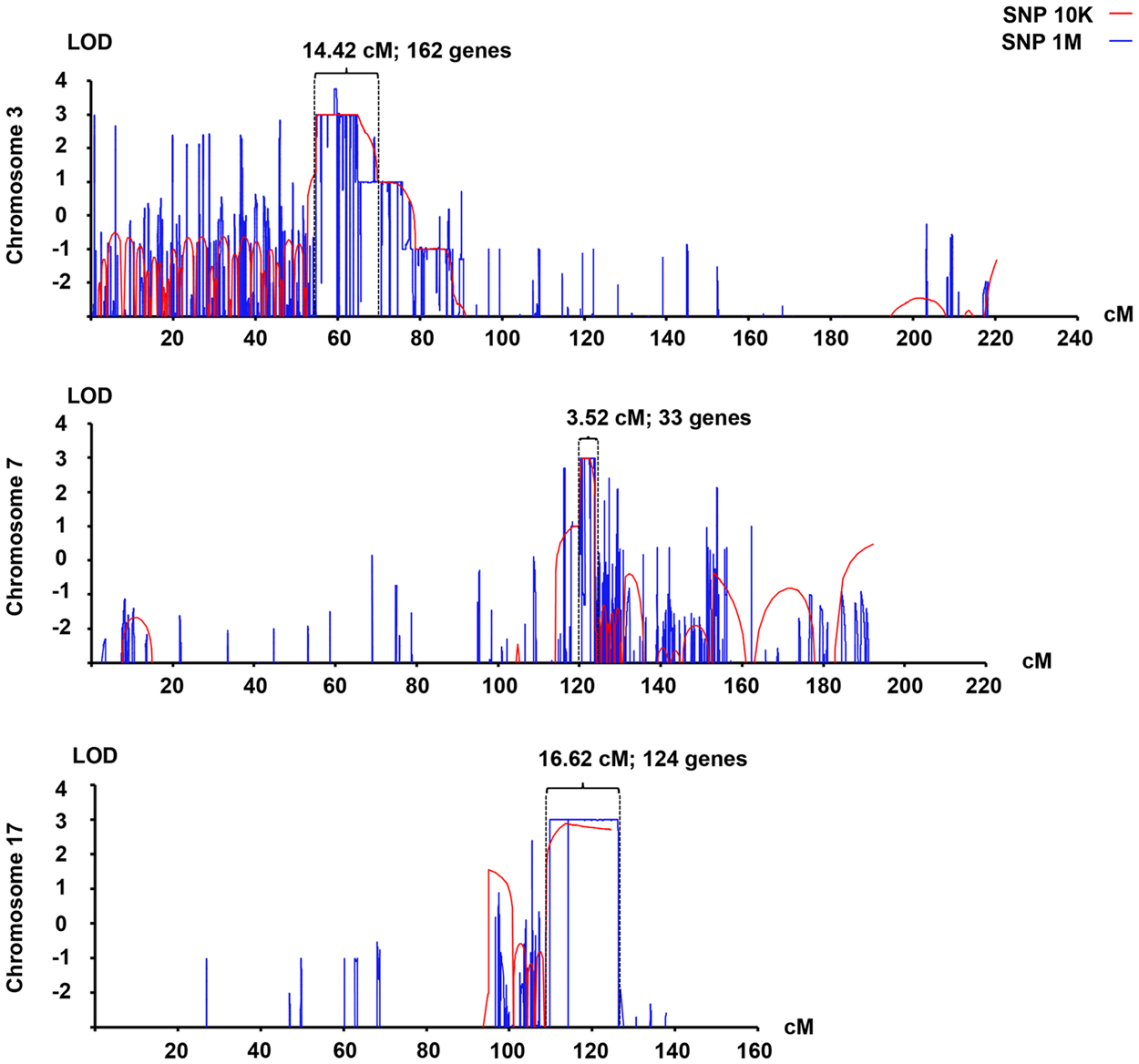
Linkage intervals on chromosomes 3, 7 and 17. The genome-wide linkage analysis of the UBRS082 family was carried out by using either SNP 10 K array (Human Mapping 10 K 2.0; Affymetrix, USA) and SNP 1 M array (Genome-Wide Human SNP Array 6.0; Affymetrix, USA), and analyzed by using easyLINKAGE software, set as autosomal dominant model in multi-point parametric mode (the analyses in other modes are not shown). The regions with high log of odd (LOD) scores on chromosomes 3, (Max LOD = 3.75), chromosome 7 (Max LOD = 3.01), and chromosome 17 (Max LOD = 2.98) were located.

The SNP-genotyping datasets from either SNP 10 K or SNP 1 M microarrays of this family were analyzed by using easyLINKAGE software^17^, in the autosomal dominant model. The regions with Max LOD scores analyzed by two-point or multi-point parametric mode or in non-parametric mode are related to each other (Supplementary Table S1). We selected the continuous regions from the Max LOD scores of multi-point parametric mode on chromosomes 3, (Max LOD = 3.75), chromosome 7 (Max LOD = 3.01), and chromosome 17 (Max LOD = 2.98) for further analyses (Fig. 2). These three chromosomal regions covered the intervals of 15.2 Mb, 6.3 Mb, and 5.8 Mb, respectively (Table 2).

**Table 2:** Numbers and types of genetic variations within three high LOD regions on chromosomes 3, 7, and 17.

Chromosome [position]	Size (Mb)	No. variations	No. exonic variations	No. exonic variations in affected (dominant model)			
				Synonymous	Non synonymous	In/Del	Stop gain/loss
3 [30, 303, 774–45, 507, 305]	15.2	881	170	7	13	0	0
7 [111, 578, 521–117, 907, 989]	6.3	166	21	0	0	0	0
17 [71, 726, 922–77, 477, 826]	5.8	885	183	11	6	0	0
Total	27.3	1,932	374	18	19	0	0

DNA samples from two affected members (II:5 and II:6) and one unaffected member (II:7) were selected for exome capturing and sequencing (Illumina, USA). Genetic variations in the candidate regions on chromosomes 3, 7 and 17 in these three family members, containing 1,932 variations with the average read depth of 38x, were filtered by exclusion of synonymous variations but selection for non-synonymous, stop, gain/loss, and short insertion or deletion (In/Del) variations. After the filtration, 19 variations remained for further analysis (Table 2).

The impact of amino acid changes on the protein structures and functions of the 19 candidate variations were predicted by using 6 web-based programs. Four variations, including two rare variations in *SCN10A* [GenBank: NM_006514.3; c.2727 C > A (p.N909K) and c.5426 A > G (p.K1809R) (MIM: 604427)] (rs567269429 and rs561166361), one reported variation in *XIRP1* [GenBank: NM_194293.2; c.4501 G > A (p.V1501M) (MIM: 609777)] (rs58805228), and one rare variation in *TTYH2* [GenBank: NM_032646.5; c.531 G > A (p.M177I) (MIM: 608855)] (rs371502920) were predicted as disease-causing or damaging by at least 3 of 6 programs (Supplementary Table S2).

### Genotyping of genetic variations in family members and normal control subjects

The four variations predicted to be pathogenic or damaging were genotyped in all affected and unaffected members of the index family. All variations co-segregated with KSD in the family (Supplementary Table S3). These four variations were further tested in DNA samples from normal control subjects (n = 180), living in the same geographical area, by PCR-HRM analysis (Supplementary Fig. S1). Two variations in *SCN10A* (p.N909K and p.K1809R) were not present in the 180 normal control subjects tested while the two variations in *XIRP1* (p.V1501M) and *TTYH2* (p.M177I) were observed in 24 and 3 normal control subjects, respectively (Supplementary Table S3). Furthermore, *TTYH2* (p.M177I) was present but did not co-segregate with KSD in 4 affected families (Supplementary Fig. S2). Thus, the variations in *XIRP1* (p.V1501M) and in *TTYH2* (p.M177I) were excluded as they might not be implicated in KSD. However, the two variations in *SCN10A* (p.N909K and p.K1809R) showed their segregation in this family, suggesting that *SCN10A* was involved in KSD in this family. Haplotype analysis suggested that the allele with two variations was inherited from the father (I:1) who was deceased (Fig. 1A and Supplementary Fig. S3).

### Mutation screening and analysis of genetic variations

We genotyped these two variations and screened all 27 exons of *SCN10A* in the DNA samples from 180 patients with KSD by the PCR-HRM and Sanger sequencing. A total of 29 variations in *SCN10A* (1 novel and 28 reported variations) were identified (Supplementary Fig. S4 and Supplementary Table S4). The functional impacts of all exonic variations were analyzed as previously mentioned (Supplementary Table S5) and the impact of exon-intron boundaries on mRNA splicing process was evaluated by using ESE finder 2.0^18^. We found a variation in *SCN10A* (p.V1149M) associated with KSD in another family (UBRS094) (Fig. 1B), which was not present in the normal control subjects (n = 180). The clinical characteristics of KSD in the affected members of this family are shown in Supplementary Table S6.

### mRNA and protein expression, immunohistochemistry, and double immunofluorescence staining in human kidney tissues

**Figure 3:**
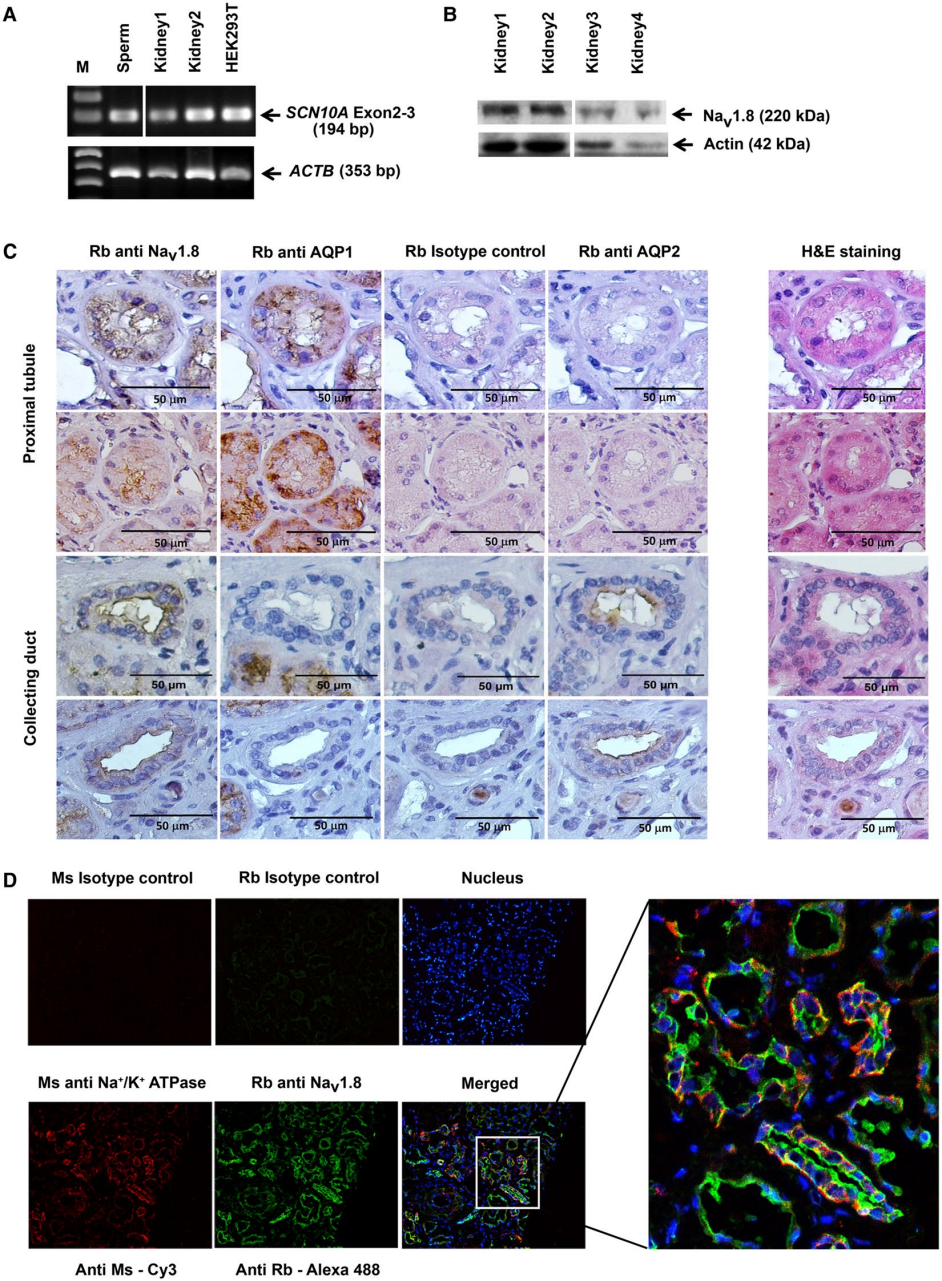
Expression of *SCN10A* mRNA and Na_V_1.8 α subunit protein in human kidney tissues. (**A**) Detection of *SCN10A* mRNA from human kidney tissues and HEK293T by RT-PCR method. *SCN10A* mRNA extracted from sperm was used as a positive control. A region of *SCN10A* mRNA covering exons 2–3 was analyzed, and mRNA of a house-keeping gene– *ACTB* was used as an internal control. (**B**) Western-blot analysis showed Na_V_1.8 α subunit of voltage-gated sodium channel expression in four human kidney tissue samples. Actin was used as loading control. (**C**) Detection of Na_V_1.8 α subunit protein by immunohistochemistry. The protein was stained in proximal tubules (2 upper panels in the first column) as well as collecting duct (2 lower panels in the first column) of human kidney. AQP1 (second column) was used as protein marker of proximal tubule and AQP2 (fourth column) as protein marker of collecting duct. Rabbit antibody was used as isotype control (third column). Hematoxyline and eosin staining of kidney tissue is shown in the fifth column. The original magnification was 40×. (**D**) Detection of Na_V_1.8 α subunit protein by double immunofluorescene staining in human kidney tissue. Fresh frozen human kidney-tissue section was incubated with mouse (Ms) or rabbit (Rb) antibody as isotype control (the first and second top panels), incubated with Hoechst 33258 for nuclear staining (blue), and incubated with mouse (Ms) anti-alpha 1 Na^+^/K^+^ ATPase antibody (red) for membrane staining (the first bottom panel) or rabbit (Rb) anti-Na_V_1.8 antibody (green) for Na_V_1.8 α subunit protein staining (the second bottom panel). The first and second bottom panels were merged in the third bottom panel and then enlarged. The original magnification was 20×. Images of the gels and blots cropped from different parts of the same gel/blot, or from different gels/blots were separated by white space. The full-length gels and blots are presented in Supplementary Fig. S9.

*SCN10A* mRNA and Na_V_1.8 α subunit protein in human kidney tissues were examined by RT-PCR and Western-blot analysis as shown in Fig. 3A,B, respectively. *SCN10A* mRNA was found to express in human kidney tissues and HEK293T cell line (Fig. 3A and Supplementary Fig. S5). The protein was observed in all 4 human kidney tissues (Fig. 3B). The Na_V_1.8 α subunit protein was then stained by immunohistochemistry (Fig. 3C) and double immunofluorescence methods (Fig. 3D). The protein was expressed at the proximal tubule and the collecting ducts of nephrons (Fig. 3C), and its expression at tubules of nephron co-localized with alpha 1 Na^+^/K^+^ ATPase, a membrane protein marker of the kidney tubules (Fig. 3D).

### Quantitative analyses of wild-type and mutant Na_V_1.8 α subunit proteins expressed in HEK293-1B cells

**Figure 4:**
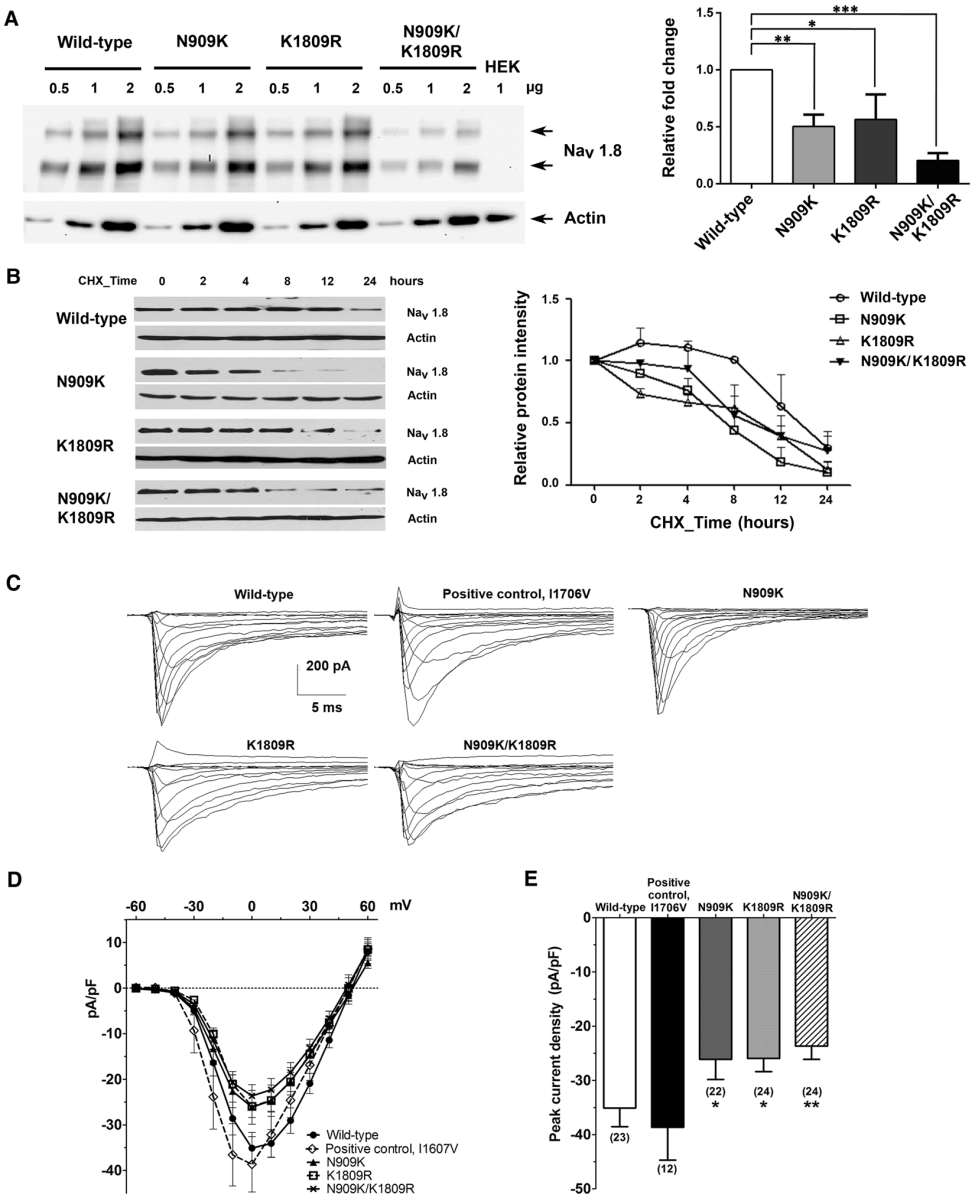
Effect of *SCN10A* mutations on the protein expression in transfected HEK293-1B cells and electrophysiological properties. (**A**) Semi-quantitative Western-blot analysis and quantitative representation of wild-type and mutant Na_V_1.8 α subunit proteins and endogenously expressed actin. The mutant proteins had decreased amounts compared to that of the wild-type. The bar graph shows staining densities of the proteins normalized by that of actin. Results are shown as means ± SEM of the relative signal intensities from three independent experiments. Statistical analysis was performed by using the Sidak’s multiple comparison test: *p < 0.05, **p < 0.01, ***p < 0.001. (**B**) Stabilities of wild-type and mutant Na_V_1.8 α subunit proteins in transfected HEK293-1B cells after treatment with 100 μg/ml of cycloheximide (CHX) for 0, 2, 4, 8, 12 and 24 hours. The wild-type and mutant proteins were quantified by GeneTools software version 4.03 (Syngene, UK) and plotted as relative intensities. The data are shown as means ± SEM from three independent experiments. (**C**) Representative wild-type and mutant Na_V_1.8 currents recorded from HEK293-1B cells. Cells were held at −80 mV and stepped to membrane potentials from −60 to +60 mV for 100 ms in 10 mV increments. (**D**) Peak current density-membrane voltage relationship for wild-type and mutant Na_V_1.8 channels. (**E**) The average peak current densities at 0 mV were significantly decreased for mutant channels compared to wild-type channels. *p < 0.05 vs control, unpaired t-test. Images of the blots are cropped from different parts of the same blot. The full-length blots are presented in Supplementary Fig. S10.

HEK293-1B cells transfected with wild-type or mutant Na_V_1.8 construct were cultured for 48 hours. The expression of wide-type or mutant Na_V_1.8 α subunit protein was determined by Western-blot method and quantified by densitometric analysis. The levels of all mutant proteins significantly decreased when compared to that of the wild-type protein as analyzed by one-way ANOVA (Fig. 4A). There were no significant differences of the recombinant protein quantities between the N909K and K1809R mutants, whereas the recombinant protein from N909K/K1809R mutant showed a significant reduction when compared to those of the wild-type and the two individual mutants.

### Stabilities of wild-type and mutant Na_V_1.8 α subunit proteins expressed in HEK293-1B cells

To examine stabilities of the wild-type and mutant Na_V_1.8 proteins expressed in HEK293-1B cells, newly synthesized protein were inhibited with cycloheximide – a protein synthesis inhibitor. The level of wild-type Na_V_1.8 α subunit was initially constant and then reduced by 50% after cycloheximide treatment for more than 12 hours (Fig. 4B). In contrast, the levels of mutant Na_V_1.8 α subunit proteins (N909K, K1809R, and N909K/K1809R) were more rapidly reduced than that of the wild-type Na_V_1.8 α subunit protein. The time leading to a 50% reduction for the mutant N909K protein was more than 6 hours and for the mutant K1809R and N909K/K1809R proteins were more than 10 hours. They seemed to have time-dependent reduction within 2–24 hours (Fig. 4B).

### Electrophysiological properties of wild-type and mutant Na_V_1.8 proteins expressed in HEK293-1B cells

HEK293-1B cells were transfected to express wild-type or mutant Na_V_1.8 α subunit proteins and the electrophysiological properties of Na_V_1.8 currents were compared. Figure 4C shows representative currents, with average current density-voltage curves shown in Fig. 4D. Peak current densities at 0 mV were compared in Fig. 4E. The currents produced by mutant Na_V_1.8 p.I1706V (−38.66 ± 6.06 pA/pF, n = 12), serving as a positive control, had an average peak current density that did not differ from that of wild-type (−35.10 ± 3.44 pA/pF, n = 23). The average peak current densities of mutant Na_V_1.8 p.N909K (−26.12 ± 3.72, n = 22), p.K1809R (−25.96 ± 2.44, n = 24) and p.N909K/K1809R (−23.68 ± 2.46, n = 24) were significantly reduced, compared to that of the wild-type Na_V_1.8. The reversal potential was unaffected by all the mutations (Table 3), indicating a preservation of ionic selectivity.

**Table 3:** Reversal potential together with parameters of activation, steady-state fast inactivation, and slow inactivation of wild-type and mutant Na_V_1.8 current expressed in HEK293-1B cells.

Na_V_1.8	Reversal potential		Activation (mV)			Steady-state fast inactivation			Slow inactivation		
	mV	n	V_1/2_	*k*	n	V_1/2_	*k*	n	V_1/2_	*k*	n
Wild-type	53.02 ± 1.61	23	−5.97 ± 1.93	10.80 ± 1.00	23	−49.14 ± 1.67	10.03 ± 1.07	23	−58.23 ± 2.86	10.32 ± 0.62	12
I1706V	49.39 ± 2.05	12	−13.23 ± 2.54*	7.51 ± 1.23*	12	−47.84 ± 2.02	10.03 ± 0.70	12	−58.60 ± 4.06	11.34 ± 1.22	7
N909K	49.84 ± 1.87	22	−9.43 ± 2.09	8.43 ± 0.45	22	−48.44 ± 1.42	10.25 ± 0.54	22	−56.87 ± 1.57	10.20 ± 0.85	16
K1809R	49.37 ± 1.63	24	−4.51 ± 2.14	11.41 ± 1.15	24	−50.72 ± 2.52	10.44 ± 0.53	22	−58.47 ± 2.74	12.24 ± 0.54*	11
N909K/K1809R	48.29 ± 1.83	24	−6.56 ± 2.37	10.77 ± 1.05	24	−47.24 ± 2.02	10.44 ± 0.48	23	−56.12 ± 2.36	11.75 ± 0.81	16

Mean ± S.E.M.; V_1/2_, half maximal activation or inactivation voltage; k, slope factor; *p < 0.05 versus wild-type channels (unpaired t-test).

The Boltzmann fit for activation, fast inactivation, and slow inactivation were derived for each individual cell and averaged (Table 3). The voltage dependence of activation is illustrated in Supplementary Fig. S6 (G/G_max_, right curves of each panel). The activation midpoint (V_1/2_) of mutant Na_V_1.8 p.I1706V has significantly shifted to more hyperpolarized potentials with steepened slope factor (k) than wild-type (Table 3 and Supplementary Fig. S6A). On the other hand, the mutant Na_V_1.8 p.N909K, p.K1809R and p.N909K/ K1809R had no significant effect on activation or steady-state fast inactivation (Table 3 and Supplementary Fig. S6B–D). Mutant Na_V_1.8 p.N909K and p.N909K/K1809R had no significant effect on slow inactivation, but mutant Na_V_1.8 p.K1809R had a slight increased slope factor than the wild-type (Table 3).

We further investigated the kinetics of fast inactivation. Fitting the current decay phase with a monoexponential fit revealed that fast inactivation rates were similar between wild-type and mutant Na_V_1.8 proteins, except for the mutant Na_V_1.8 p.K1809R which had significantly increased time constant (Fig. 4C and Supplementary Table S7). At 0 mV, the average percentage of persistent current (caused by slow inactivation^19^) was significantly reduced in mutant Na_V_1.8 p.I1706V and p.N909K compared with wild-type (Supplementary Table S7), while that of mutant Na_V_1.8 p.K1809R and p.N909K/K1809R was not (Supplementary Table S7). Finally, we examined the rate of recovery from fast inactivation at −80 mV, using a two-pulse protocol (see the Methods), and found that among the wild-type and mutant Na_V_1.8 channels, there was no difference in recovery time constants of either slow or fast component (Supplementary Fig. S7 and Supplementary Table S8).

## Discussion

The etiology of KSD in the NE Thai population is unknown; it is possibly different from what was reported in the Western and other populations because there are no hypercalciuria, hyperoxaluria or hyperuricosuria observed in the affected persons in this population^20^. Previously, our group has demonstrated that genetic variations were associated with KSD risk in the NE Thai population by case-control studies. We found that genetic variation of a stone inhibitor protein (prothrombin, F2) was associated with KSD risk in the NE Thai female patients^15,16^ and later reported the association between a 3′ UTR variation of *PAQR6* – a gene encoding progestin and adipoQ receptor family member VI, and KSD in the NE Thai patients^21^. However, these genetic variants were likely modifying, not disease-causing, variants because the minor alleles were observed in both the patient and control groups with different frequencies. In contrast with the other ethnic groups, the genes associated with hypercalciuria such as *VDR*, *CLCNS*, *CLDN16*, *TRPV5* and *KLOTHO*^22^ were not detected in the NE Thai patients. We therefore set forth to identify a disease-casing gene in the NE Thai families with KSD by using linkage analysis and exome sequencing.

We initially identified three Max LOD regions in an index family affected with KSD by genome-wide linkage analysis and found two variations in *SCN10A* (p.N909K and p.K1809R) co-segregated with KSD in this family (Fig. 1A). Haplotype analysis in the chromosome 3 region, where the two variations (p.N909K and p.K1809R) and 49 SNPs in *SCN10A* located, showed co-segregation of one haplotype with KSD in this family (Supplementary Fig. S3). The two variations of *SCN10A* (p.N909K and p.K1809R) are thus in linkage disequilibrium and their effects to cause the disease may be combined. The combined amino-acid changes may exert a greater effect upon structural alteration of the protein. Combinations of two mutations on the same allele have previously been reported in other diseases inherited as autosomal dominant mode such as a missense (p.N543H) mutation and an in-frame 9-bp deletion (2393del9) of *low-density lipoprotein receptor* gene in familial hypercholesterolaemia^23^ and missense mutations at p.V804M and p.Y806C of *RET* proto-oncogene in multiple endocrine neoplasia type 2B (MEN2B)^24,25^. Both studies showed that the combinations of two mutations on the same alleles could act together to increase effect on the protein function.

An additional variation of *SCN10A* (p.V1149M) identified was potentially associated with KSD in another affected family (Fig. 1B). The discovery of another variation of *SCN10A* (p.V1149M) associated with KSD in a different family strongly supports that this gene is involved in KSD. *SCN10A* encodes Na_V_1.8 α subunit of voltage-gated sodium channel (Fig. 1C). Amino-acid sequence alignment demonstrated that asparagine (N) 909, valine (V) 1149, and lysine (K) 1809 are highly conserved in the evolution of vertebrates (Fig. 1D), supporting their functional significance in the protein structure.

The affected members of the index family are males in majority while the affected members of the second family are mainly females. Since *SCN10A* locates on chromosome 3 which is an autosome, sex preference may not be involved in the disease association with the defect of this gene, and the sex bias in each family is likely occurred by chance. However, it is possible that other genetic factors for example the presence of *F2* variations, which is a KSD modifying gene in the NE Thai population, may be involved in the gender and disease association in favor of the affected male majority.

The Na_V_1.8 α subunit of voltage-gated sodium channel encoded by *SCN10A* plays a key role in nociception – the neural processes of encoding and processing noxious stimuli to signal pain^26^. Na_V_1.8 α subunit is expressed in dorsal root ganglion (DRG) neurons and peripheral nerve axons^27^. Gain-of-function mutations of *SCN10A* cause painful peripheral neuropathy^19,28,29^. Na_V_1.8 α subunit is also expressed in heart tissue and genetic variation in *SCN10A* associated with prolonged cardiac conduction and Brugada syndrome^30–32^. However, these clinical phenotypes were not observed in the affected members of the studied families. The absence of a primary role for *SCN10A* mutations in arrhythmogenic right ventricular dysplasia/cardiomyopathy was reported and it has previously been noted that despite a known role for the encoded protein in peripheral nerve function, the proband with the *SCN10A* mutation had no discernible neurological abnormalities^33^. Thus, it is likely that the affected family members with KSD associated with *SCN10A* mutations have neither neurological nor cardiac abnormalities.

The distinct disease phenotypes associated with *SCN10A* mutations may be explained by different types of amino acid changes and different interactions with other proteins in different cell types, i.e. neurons, cardiac cells or kidney cells. For examples, while gain-of-function mutations of *SCN10A* cause painful peripheral neuropathy, its loss-of-function mutations result in prolonged cardiac conduction disease and Brugada syndrome or KSD. The two latter diseases associated with loss-of-function mutations may subtly differ in locations, types, and effects of amino acid changes on the protein structure that cause abnormality in different cell types and organs. In the prolonged cardiac conduction disease and Brugada syndrome, the mutations seem to be more severe than that in KSD. It should be noted that in the northeastern part of Thailand not only KSD but also sudden unexplained nocturnal death syndrome (SUNDS) are prevalent. Since KSD and SUNDS are associated with channelopathies but the cause of SUNDS in the NE Thai population has not fully been elucidated, it is also interesting to investigate the causal relationship between SUNDS and other mutations of *SCN10A*.

*SCN10A* mRNA and Na_V_1.8 α subunit protein were present in human kidney tissues (Fig. 3A,B and Supplementary Fig. S5). Low level of *SCN10A* mRNA expression in human kidney has previously been demonstrated by quantitative RT-PCR^34^ but there was no report on the Na_V_1.8 α subunit protein expression in the human kidney. Na_V_1.8 α subunit protein expression in proximal tubules and collecting ducts of nephron in human kidney was demonstrated by immunohistochemistry and immunofluorescence staining (Fig. 3C,D). Na_V_1.8 was initially reported to be preferentially expressed in peripheral sensory neurons and absent from kidney tissues of rodents^27^. These data are not easily reconcilable with the genetic, biochemical and functional evidences presented in this study, describing the Na_V_1.8 expression in human kidney tissue and its contribution to kidney stone disease. One explanation is that the Na_V_1.8 expression in human kidney is different than in rodents. The level of Na_V_1.8 expression and its role in kidney cells might be investigated in more detail in future studies using induced pluripotent cells that could be differentiated into kidney cells *in vitro*^35^.

The biological properties of wild-type and mutant Na_V_1.8 α subunit proteins (p.N909K, p.K1809R, and p.N909K/K1809R) were examined in HEK293-1B cells. In the quantitative Western-blot analysis, the relative fold changes of each individual mutant Na_V_1.8 α subunit protein (p.N909K or p.K1809R) and double mutant Na_V_1.8 α subunit protein (N909K/K1809R) were lower than that of the wild-type protein (Fig. 4A). These results suggested that the mutations affect the levels of Na_V_1.8 α subunit protein expression. To investigate their stabilities, we then inhibited the newly synthesized proteins by cycloheximide (Fig. 4B). All mutant Na_V_1.8 α subunit proteins were more rapidly decreased than that of the wild-type protein, although the levels of the two mutant proteins (p.N909K and p.K1809R) and that of the double mutant protein (p.N909K/K1809R) were not different, suggesting the instability of the mutant proteins attributable to amino acid changes.

The electrophysiological function showed that the current density of three mutations, Na_V_1.8 p.N909K, p.K1809R, and p.N909K/K1809R, were significantly smaller than that of the wild-type, without impairing the activation or inactivation kinetics. Moreover, both single and double substitutions resulted in similar current reduction and preservation of electrophysiological properties. Therefore, the substituted residues have affected Na_V_1.8 α subunit protein stability, as demonstrated and previously discussed, more than the voltage dependence or kinetic properties, causing reduced current density. Thus, loss-of-function in *SCN10A* mutation – the mechanism involving in KSD – is attributable to the stability of the double mutant Na_V_1.8 p.N909K/K1809R protein.

The affected members of the index family had KSD, which is likely to be calcium (oxalate) stone, whose risk factors include high sodium intake^36^, and high dietary calcium or oxalate^37^. High Na^+^ in the renal ultrafiltrate is known to reduce Ca^2+^ reabsorption and may increase the tendency for stone formation^38^. As found in this study, Na_V_1.8 channel is localized in proximal and collecting tubule epithelia. We therefore speculate that, physiologically, when Na^+^ load is high, causing depolarization in the renal tubular cells, Na_V_1.8 channel may be activated by the depolarization and help increase Na^+^ reabsorption. Thus, the loss-of-function of the mutant Na_V_1.8 p.N909K/K1809R may not allow sufficient Na^+^ reabsorption increase to match the increased Na^+^ load, resulting in high filtered Na^+^, causing decreased Ca^2+^ reabsorption and high urinary Ca^2+^. These cationic (Na^+^ and Ca^2+^) changes can be the initial risk factor of KSD caused by the loss-of-function mutations of *SCN10A*. Although these changes may not be large to alter urinary electrolytes, their effects can eventually cause observable KSD in the affected members of this family. The late-onset nature of KSD in this family, generally >25 years old, indicates the other factor, either genetic or environmental one, contributing to the pathogenesis of KSD. The additional genetic mutation at another allele occurred as somatic mutation (or second hit) in *SCN10A* or epigenetic alterations regulating *SCN10A* expression may be involved in the late-onset nature of the disease. This additional genetic mutation or epigenetic alterations may exert a greater reduction of Na_V_1.8 channel and larger cationic (Na^+^ and Ca^2+^) changes, increasing the risk of KSD. Alternatively, the known environmental factors, such as high Na^+^, Ca^2+^ or oxalate diet, or unknown environmental factors in combination with the loss-of-function mutations of *SCN10A* may contribute to the pathogenesis of KSD in this family. These additional factors are still unclear and required further studies.

Taken together, we discovered loss-of-function mutations of *SCN10A*, encoding Na_V_1.8 α subunit of voltage-gated sodium channel, in families with KSD. Two mutations (p.N909K and p.K1809R) in the same allele and one additional mutation (p.V1149M) of *SCN10A* co-segregated with KSD in two affected families. The mutant protein containing p.N909K/K1809R variations were unstable and reduced in the regulation of current density function of Na_V_1.8 channel. The loss-of-function of the mutant Na_V_1.8 p.N909K/K1809R channel may be associated with cationic (Na^+^ and Ca^2+^) imbalance by reduced Na^+^ reabsorption, resulting in high Na^+^ filtration and decreased Ca^2+^ reabsorption, which is the risk factor of KSD in the affected families.

## Methods

### Patients with kidney stone disease and normal control subjects

This study was approved by Siriraj Institutional Review Board (SIRB) and Ethical Committee of the Ministry of Public Health, Thailand. The written informed-consent was obtained from all subjects before the study. Two hundred and fifty-six patients with kidney stone disease (KSD) and their family members were recruited at Sappasithiprasong Hospital in Ubon Ratchathani Province, Thailand. The patients’ clinical and family data, blood and urine samples, and stones removed by surgery, were collected. All patients and subjects were investigated for kidney stones by radiography of kidneys, ureters, and bladder (plain KUB) and in some cases by additional ultrasonography.

The unrelated normal control subjects for this study (n = 180) were recruited from the local villagers who lived in the same geographical areas as those of the patients with KSD. All subjects were investigated by radiography (plain KUB), and confirmed with renal ultrasonography, which were found to be free from KSD, and similarly examined as that for the patients. Gender and age data of normal control subjects are shown in Supplementary Table S9.

### Genetic analysis

Genome-wide linkage analysis, exome sequencing and data analysis were performed in a selected family (UBRS082) with a maximal ELOD of 3.31, containing 17 members [7 affected and 10 unaffected]. All DNA samples were sent to the Functional Genomics Shared Resource (Vanderbilt University, Tennessee, USA) for SNP genotyping. LOD scores were analyzed by easyLINKAGE software^17^. Two DNA samples of affected members (II:5 and II:6) and one unaffected member (II:7) were taken for exome sequencing at Macrogen (Seoul, South Korea). Detailed methods are available in Supplementary methods.

Nucleotide sequences of the genes of interest were acquired from the GenBank database for designing polymerase chain reaction (PCR) primers (Supplementary Table S10). Exons were amplified using the specific primer-pairs (Supplementary Table S11), genotyped and screened in DNA samples of KSD patients and normal control subjects (see more details in Supplementary methods).

### Gene expression in human kidney and transfected proteins expressed in HEK293-1B cells

Total RNAs were extracted from human fresh frozen kidney tissues, human sperm, and HEK293T. *SCN10A* cDNA was examined by RT-PCR. The primer sequences are shown in Supplementary Table S12.

Western-blot analysis of proteins extracted from human kidney tissue, immunohistochemistry, double immunofluorescence staining, quantitative analyses and stabilities of wild-type and mutant Na_V_1.8 α subunit proteins expressed in HEK293-1B cells were examined as described in Supplementary methods.

### Electrophysiology

Transfected cells, which had been in 200 µg/ml G418 and 1 mM lidocaine for 24 hours (48 hours after transfection), were plated onto poly-l-lysine-coated cover slips (1 × 10^5^ cells per 35-mm Petri dish; lidocaine was still present), and left overnight (10–12 hours). Then, they were exposed to culture media without lidocaine for at least three hours before subjected to an electrophysiological experiment. The current density, activation, steady-state fast inactivation and slow inactivation were assessed as described in Supplementary methods.

### Statistics

Data sets were collected from at least three independent experiments. Values are expressed as mean ± SEM (standard error of the mean). One-way analysis of variance (ANOVA) followed by the Sidak’s multiple comparison test was used for statistically significant differences between the means of two groups. Differences with p < 0.05 were considered statistically significant.

### Ethics approval

Written informed consent was obtained from all studied subjects. All methods were performed in accordance with the relevant guidelines and regulations, which were approved by Siriraj Institutional Review Board (SIRB) (COA no. Si 392/2012 and COA no. Si 133/2015).

### Data availability

All the molecular genetic and cellular data generated during this study are included in this manuscript and its supplementary files. Array data are provided in Supplementary File S2 and exome sequence variant data are available at the European Variations Archive (EVA), study accession PRJEB25656.

## Electronic supplementary material

Supplementary information

Dataset 1
