# Supplementary material for: Loss-of-function mutations of SCN10A encoding NaV1.8 α subunit of voltage-gated sodium channel in patients with human kidney stone disease
Source: Sci Rep. 2018 Jul 11;8:10453. doi: 10.1038/s41598-018-28623-3 (PMC6041274; doi:10.1038/s41598-018-28623-3)
Supplement: Supplementary file 1 — Supplementary information [file 41598_2018_28623_MOESM1_ESM.pdf]

Supplementary Information

Loss-of-function mutations of *SCN10A* encoding $Na_V1.8$ α subunit of voltage-gated sodium channel in patients with human kidney stone disease

Choochai Nettuwakul[1,9], Oranud Praditsap[2,3,9], Nunghathai Sawasdee[1], Nanyawan Rungroj[2], Katesirin Ruamyod[4], Wattana B Watanapa[4], Mutita Junking[1], Sittideth Sangnual[1], Suchai Sritippayawan[5], Boonyarit Cheunsuchon[6], Duangporn Chuawattana[5], Santi Rojsatapong[7], Wipada Chaowagul[7], Sulayman D Dib-Hajj[8], Stephen G Waxman[8], and Pa-thai Yenchitsomanus[1,*]

[1] Division of Molecular Medicine, Department of Research and Development, Faculty of Medicine Siriraj Hospital, Mahidol University, Bangkok 10700, Thailand;

[2] Division of Molecular Genetics, Department of Research and Development, Faculty of Medicine Siriraj Hospital, Mahidol University, Bangkok 10700, Thailand;

[3] Immunology Graduate Program and Department of Immunology, Faculty of Medicine Siriraj Hospital, Mahidol University, Bangkok 10700, Thailand;

[4] Department of Physiology, Faculty of Medicine, Siriraj Hospital, Mahidol University, Bangkok 10700, Thailand;

[5] Division of Nephrology, Department of Medicine, Faculty of Medicine Siriraj Hospital, Mahidol University, Bangkok 10700, Thailand;

[6] Department of Pathology, Faculty of Medicine Siriraj Hospital, Mahidol University, Bangkok 10700, Thailand;

[7] Department of Medicine, Sappasithiprasong Hospital, Ubon Ratchathani 34000, Thailand;

[8] Department of Neurology & The Center for Neuroscience and Regeneration Research, Yale University School of Medicine, New Haven, CT, 06516, USA

***Correspondence:** Prof. Pa-thai Yenchitsomanus, Division of Molecular Medicine, Department of Research and Development, Faculty of Medicine Siriraj Hospital, Mahidol University, Bangkok 10700, Thailand. Email: pathai.yen@mahidol.ac.th or ptyench@gmail.com

[9] These authors contributed equally to this work.

Supplementary figures and legends

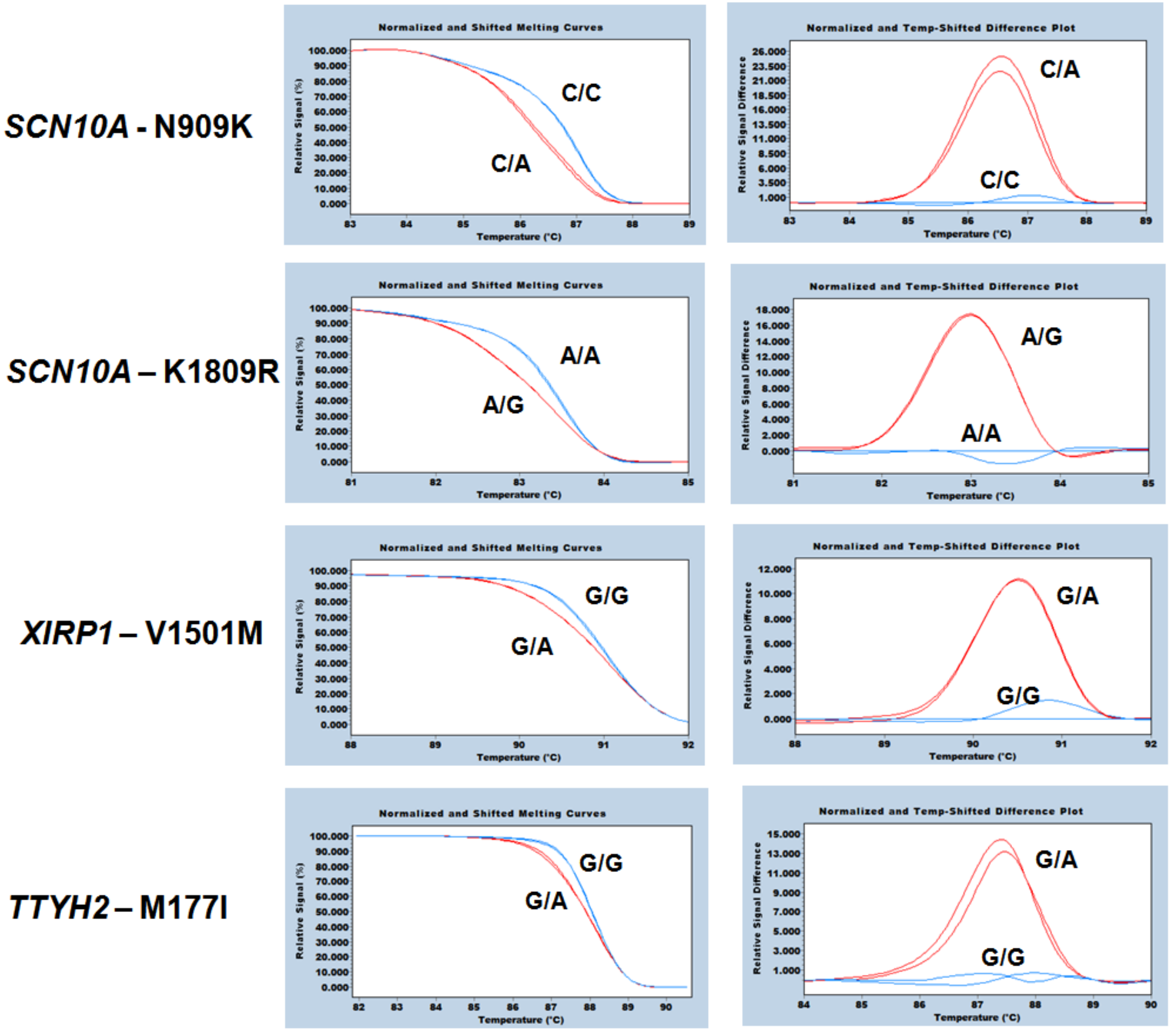

Figure S1. Genotyping of four variations, including two rare variations in *SCN10A* (rs567269429 and rs561166361; p.N909K and p.K1809R), one reported variation in *XIRP1* (rs58805228; p.V1501M), and one rare variation in *TTYH2* (rs371502920; p.M177I) by PCR-HRM method, showing melting curves and difference plots of amplicons from samples with either homozygous wild-types or heterozygous variants.

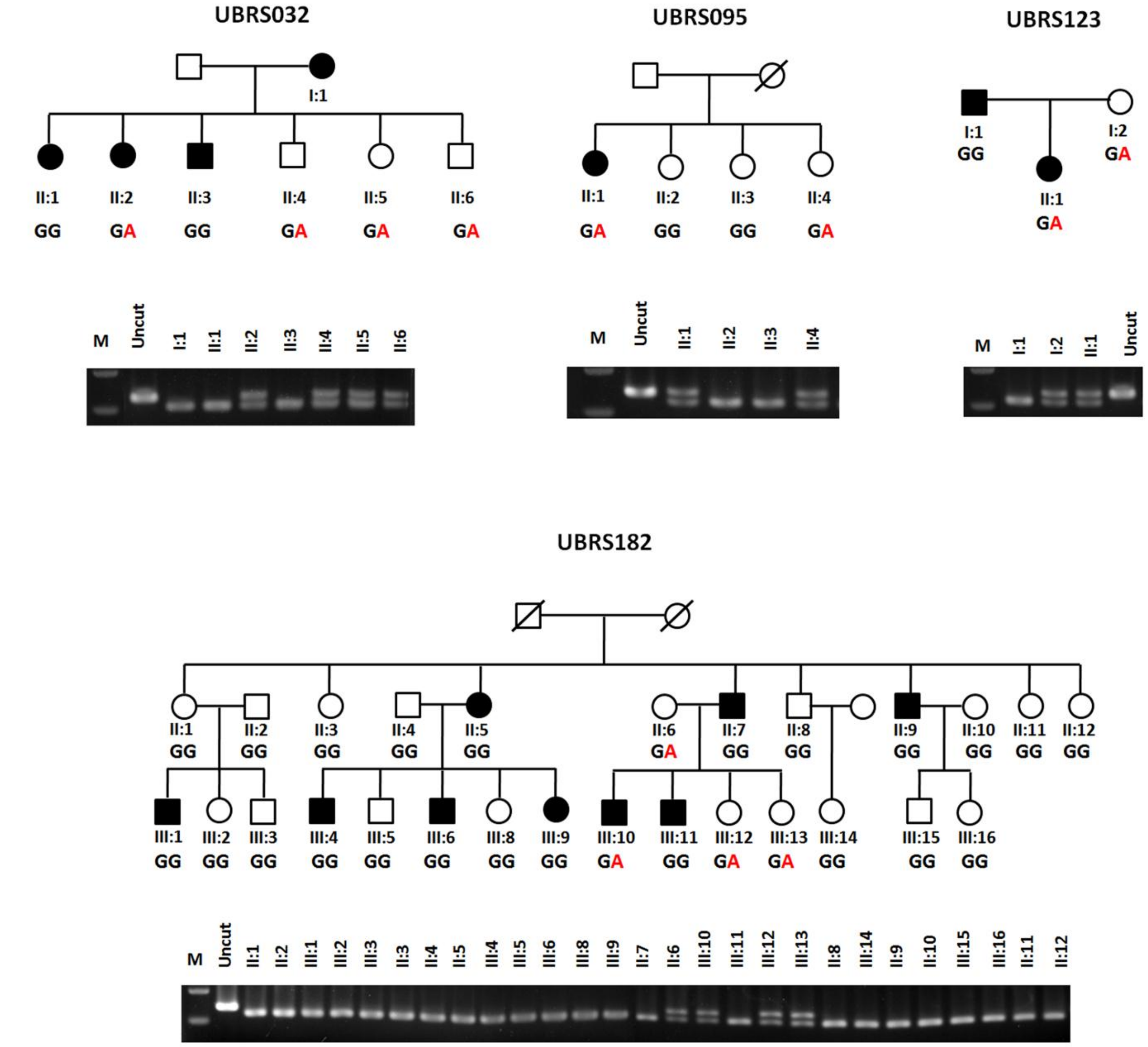

Figure S2. Segregation testing of KSD and *TTYH2* variation. p.M177I variation of *TTYH2* was genotyped by dCAPS technique in 4 affected families.

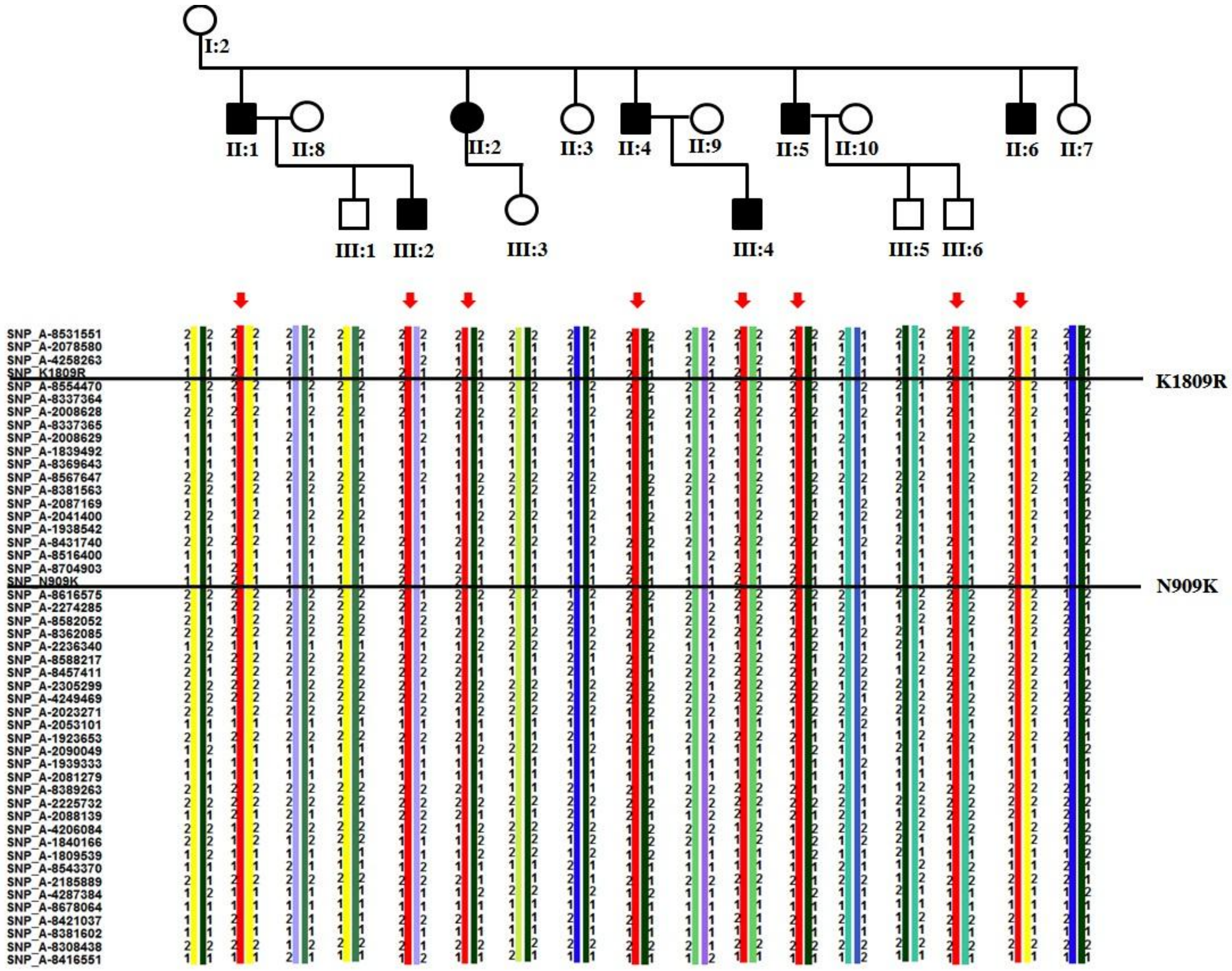

Figure S3. Haplotype analysis in the region of chromosome 3 where two variations in *SCN10A* (p.N909K and p.K1809R) and 49 single nucleotide polymorphisms (SNPs) were located, showing co-segregation of the haplotype in red color with KSD in the UBRS082 family.

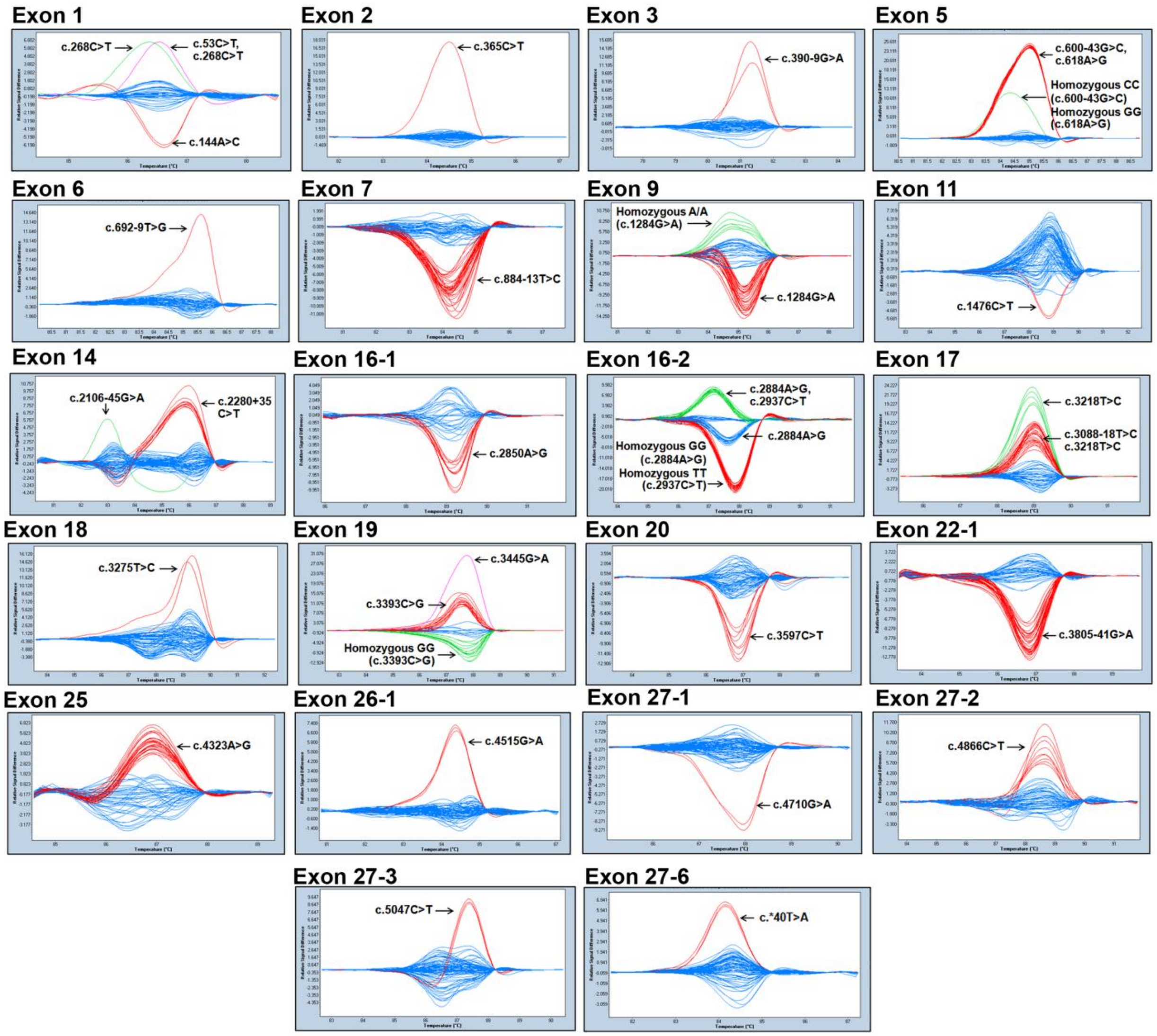

Figure S4. Screening of all 27 exons (including their exon-intron boundaries) of *SCN10A* in DNA samples from 180 patients with KSD by PCR-HRM method. Twenty-nine variations (1 novel and 28 reported variations) were identified, which were subsequently confirmed by Sanger DNA sequencing method.

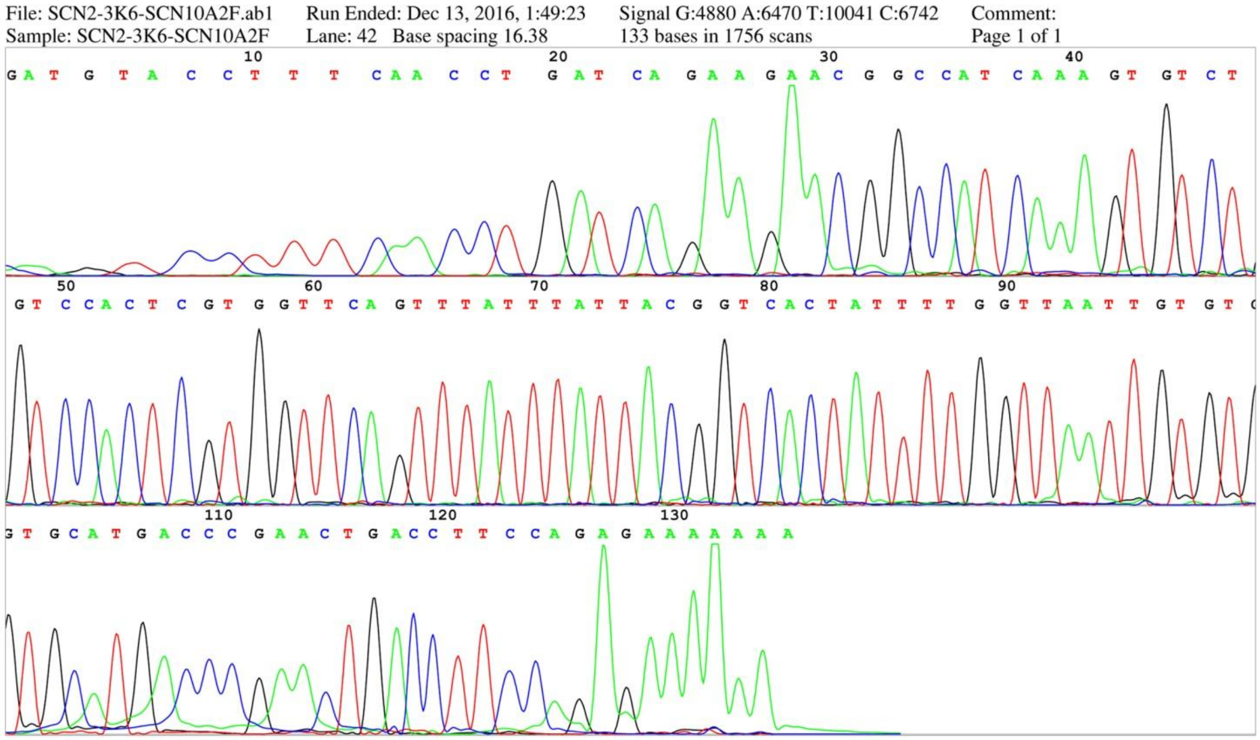

Figure S5. Sanger sequencing profile of *SCN10A* cDNA (exons 2-3), which was reversed transcribed from mRNA isolated from a human kidney tissue and amplified by polymerase chain reaction (PCR).

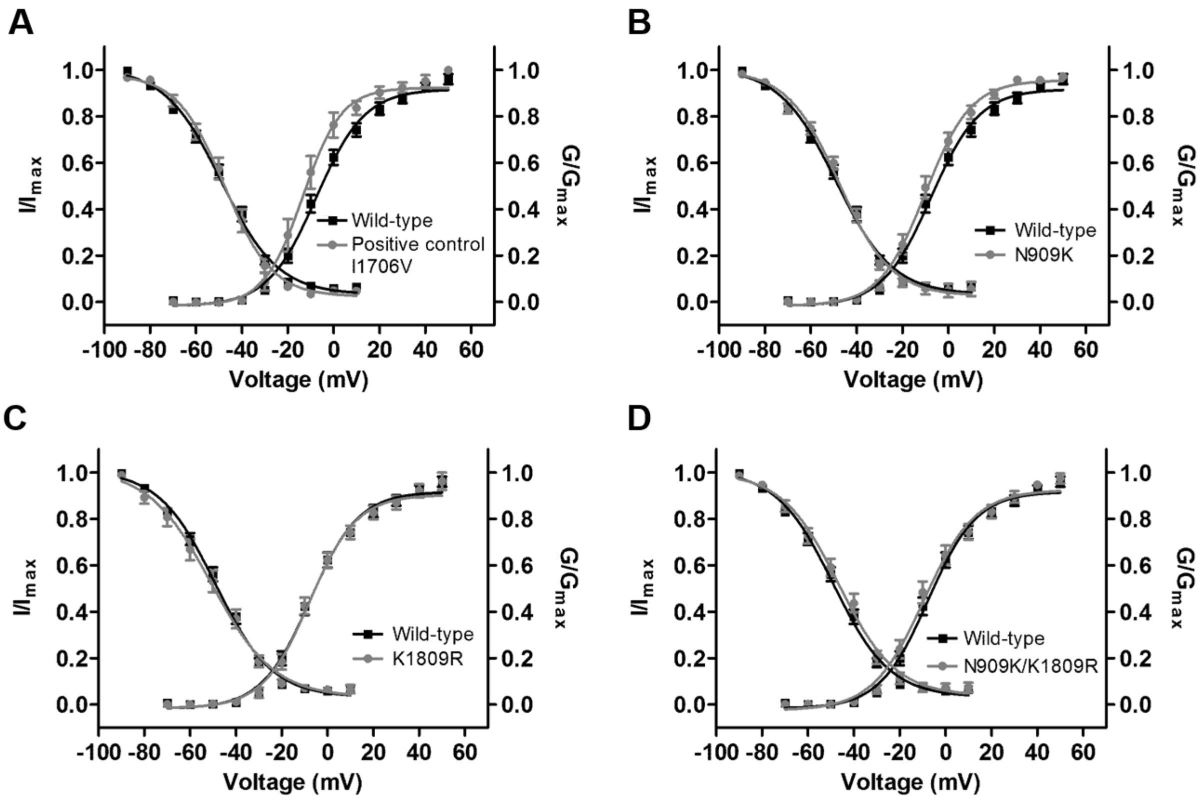

Figure S6. Comparison of the voltage dependence of activation (G/G_{max}, right curves and axis in each panel) and steady-state fast inactivation (I/I_{max}, left curves and axis) between wild-type and mutant Na_V1.8 channels; positive control (A), N909K (B), K1809R (C) and N909K/K1809R (D).

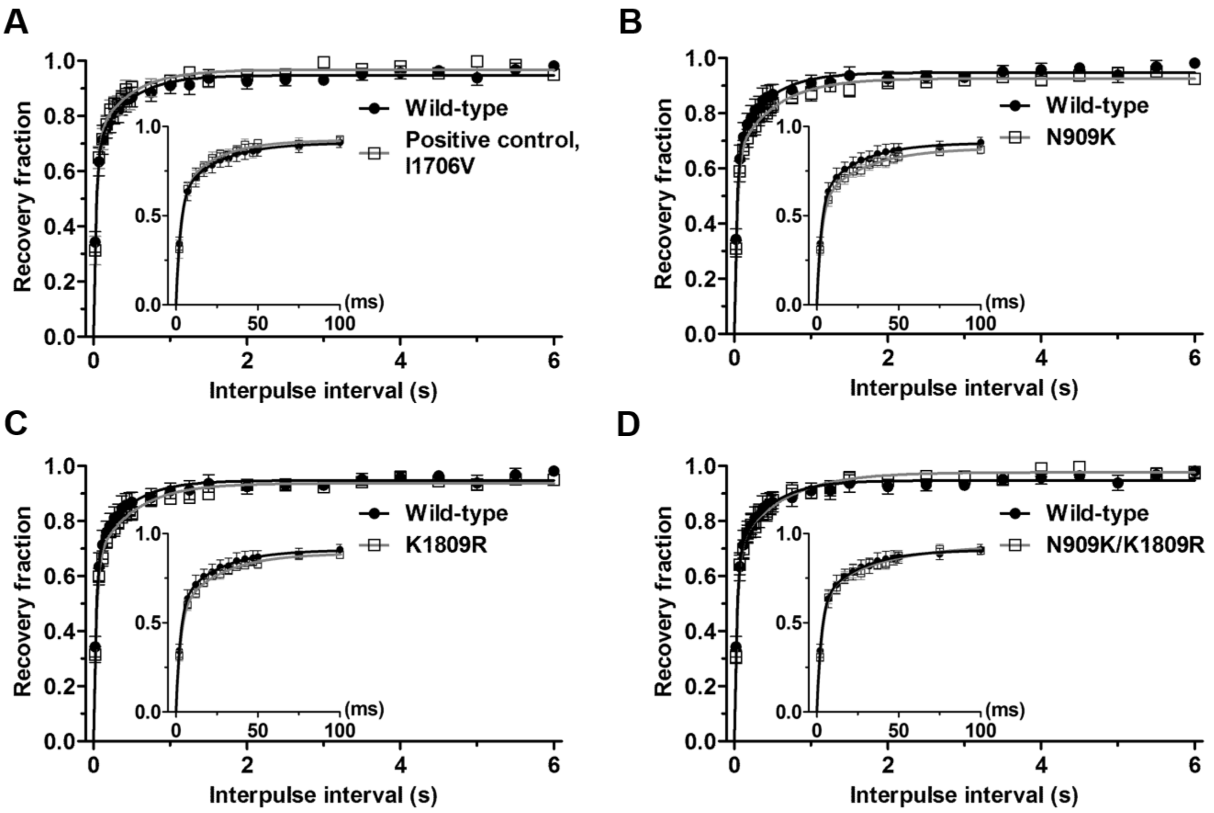

Figure S7. Time course of recovery from inactivation for wild-type and mutant $Na_V1.8$ channels at -80 mV. See Supplementary Table S8 for recovery time constant.

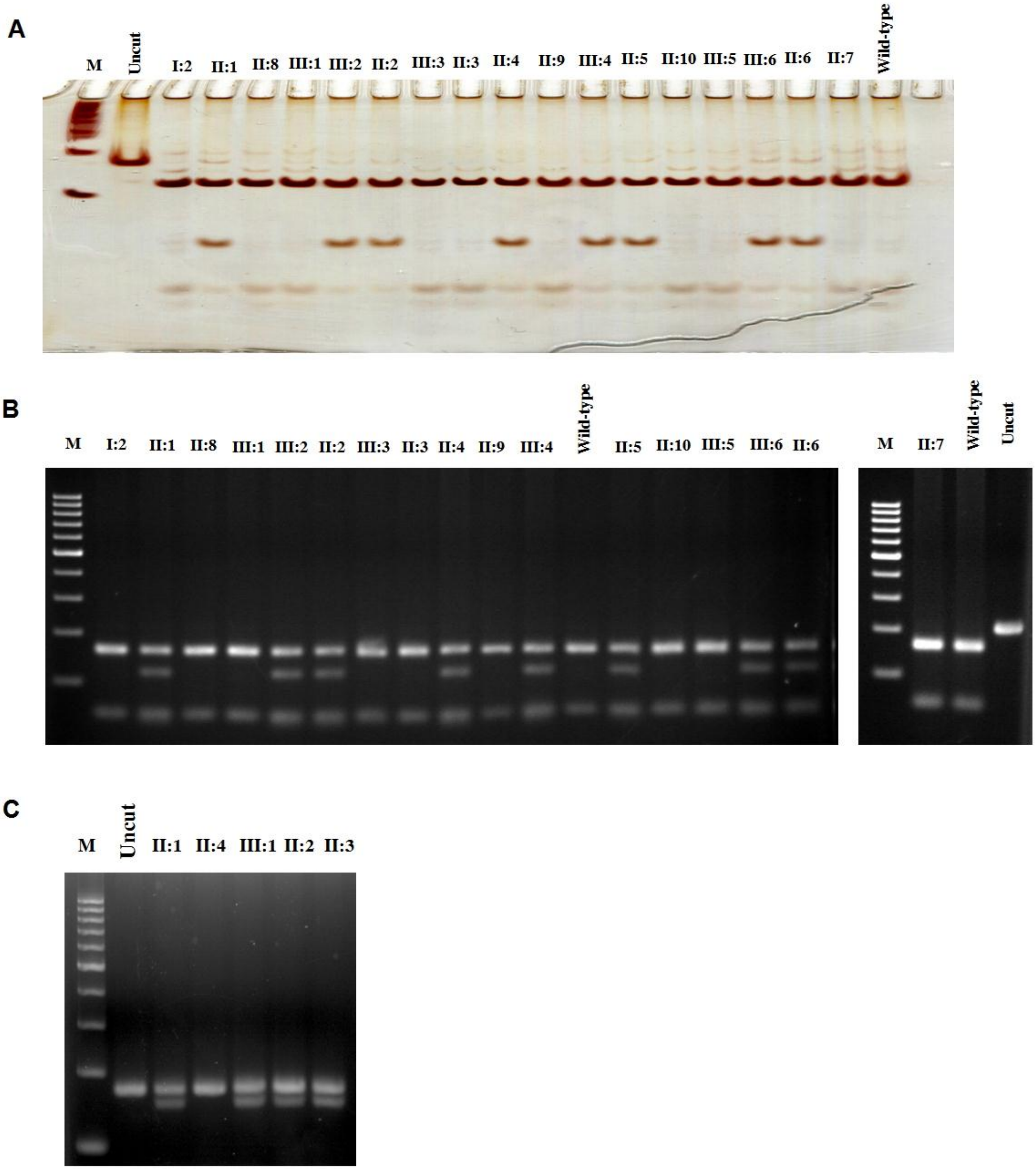

Figure S8. Segregation analysis of KSD and *SCN10A* variations in two affected families. (A) Co-segregation of KSD and *SCN10A* c.2727C>A and (B) c.5426A>G in the UBRS082 family. (C) Co-segregation of KSD and *SCN10A* c.3445G>A in the UBRS094 family.

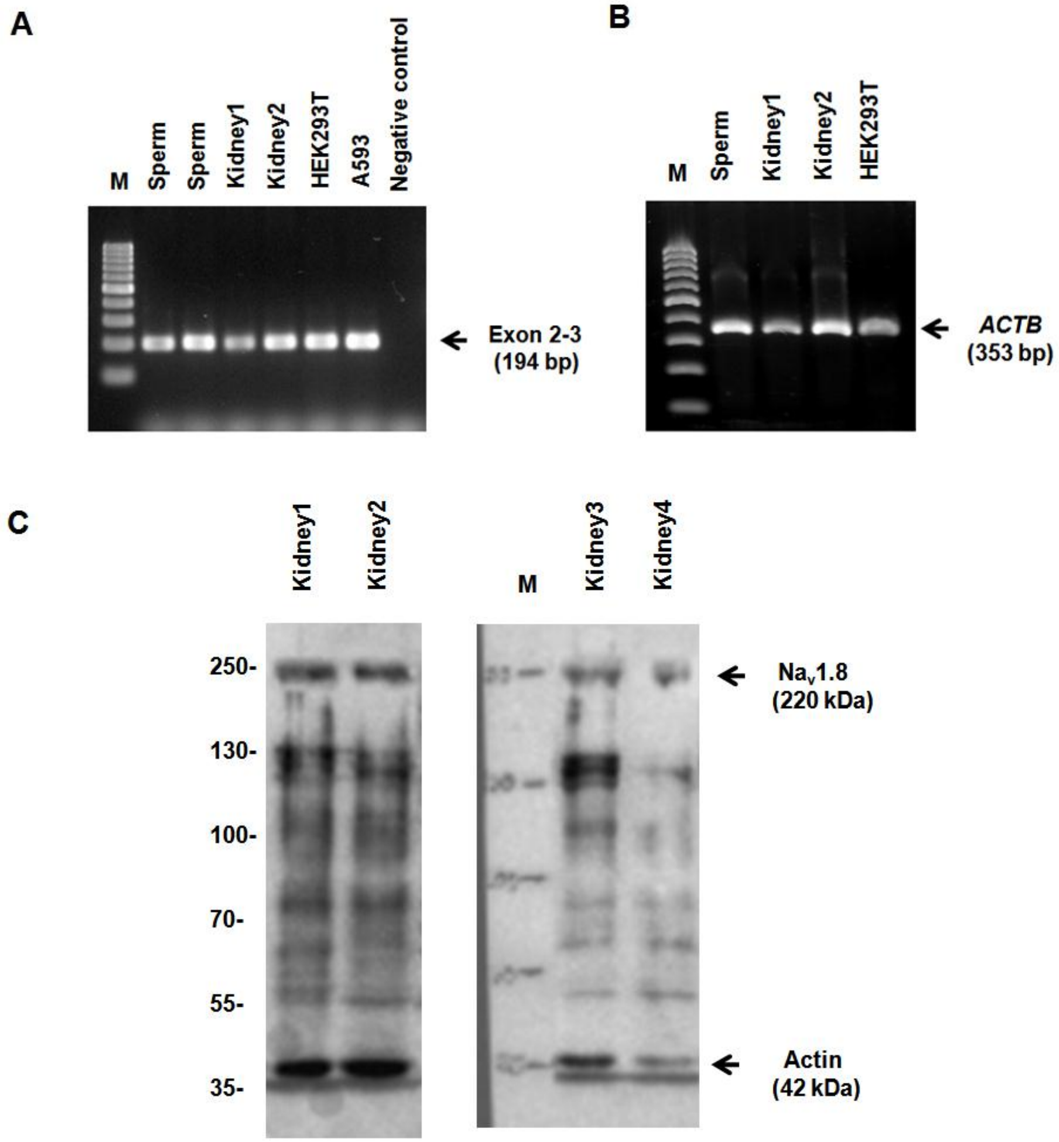

Figure S9. Expression of *SCN10A* mRNA and $Na_V1.8$ α subunit protein in human kidney tissues.

(A) Detection of *SCN10A* mRNA from human kidney tissues and cell line by RT-PCR method. *SCN10A* mRNA extracted from sperm was used as a positive control. A region of *SCN10A* mRNA covering exons 2-3 was analyzed, and (B) mRNA of a house-keeping gene– *ACTB* was used as an internal control. (C) Western-blot analysis showed $Na_V1.8$ α subunit of voltage-gated sodium channel expression in four human kidney tissue samples. Actin was used as loading control.

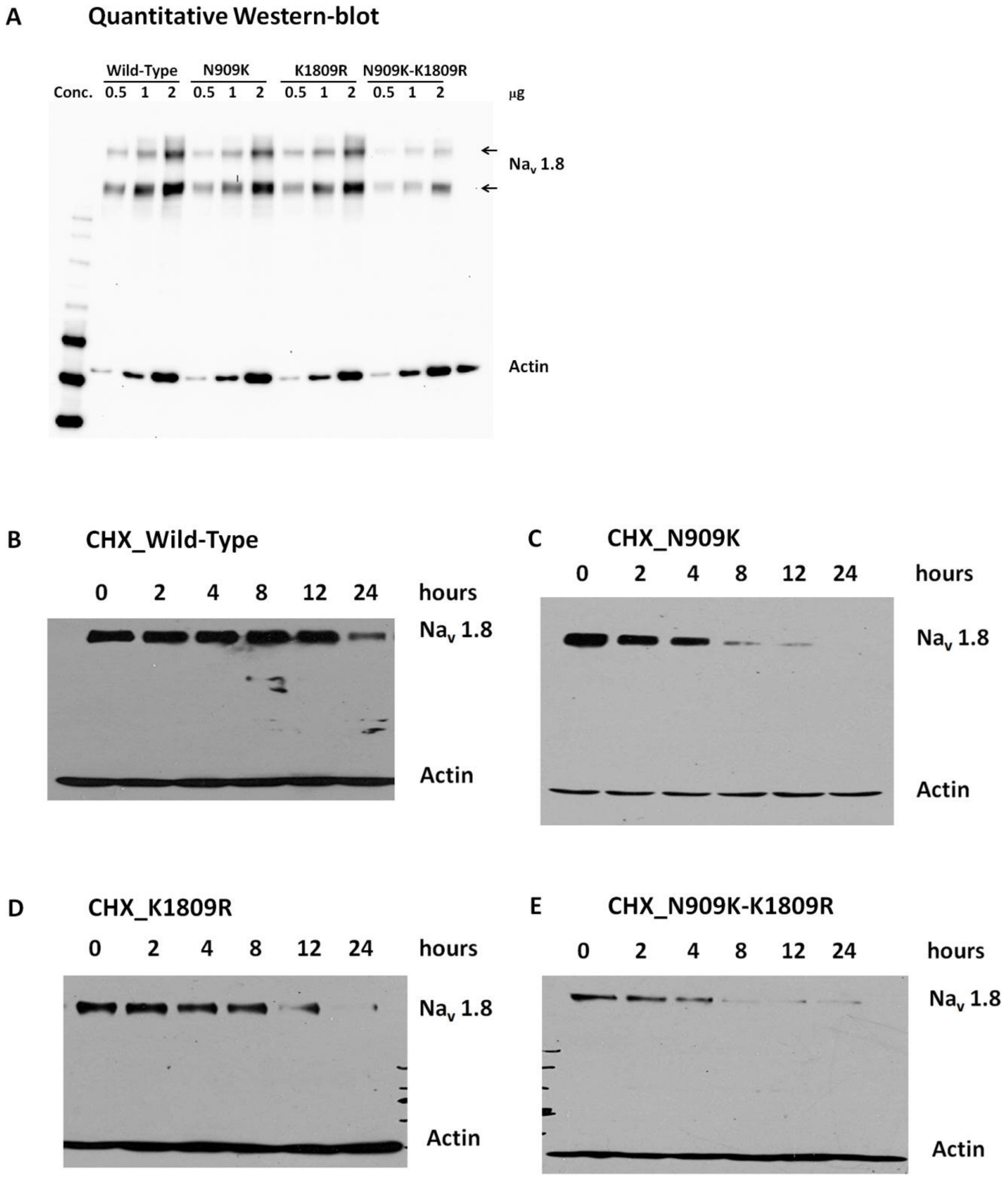

Figure S10. Effect of *SCN10A* mutations on the protein expression in transfected HEK293-1B cells.

(A) Semi-quantitative Western-blot analysis and quantitative representation of wild-type and mutant Na_V1.8 α subunit proteins and endogenously expressed actin. (B-E) Stabilities of wild-type and mutant Na_V1.8 α

subunit proteins in transfected HEK293-1B cells after treatment with 100 μg/ml of cycloheximide (CHX) for 0, 2, 4, 8, 12 and 24 hours. The wild-type and mutant proteins were quantified by GeneTools software version 4.03 (Syngene, UK).

Supplementary tables

Table S1. Results of linkage analyses showing the regions with Max LOD scores on chromosomes 3, 7 and 17.

Chr.	Two-point parametric analysis				Multi-point parametric analysis				Non-parametric analysis			
	SNP 10K		SNP 1M		SNP 10K		SNP 1M		SNP 10K		SNP 1M	
	Max LOD	Region(cM)	Max LOD	Region(cM)	Max LOD	Region(cM)	Max LOD	Region(cM)	Max LOD	Region(cM)	Max LOD	Region(cM)
3	2.99	55.31	2.98	54.86-64.44	3.01	55.26-64.83	3.75	59.20-59.79	4.35	55.31-56.55, 59.98-65.17, 69.54-69.98, 78.48-83.21	4.35	59.83-60.64, 70.67-70.72, 84.03, 93.04, 95.84
7	2.99	122.09	3.01	120.47-122.24	3.01	120.61-122.39	2.98	120.31-123.83	4.34	118.44-122.37	4.75	123.24
17	1.79	113.61	3.01	110.39-125.65	2.89	113.62-114.24	2.98	109.77-126.19	4.01	113.62	4.75	26.93, 49.81, 116.99, 120.84-126.19

Table S2. Prediction of impacts of amino acid changes on protein structures and functions of 19 candidate variations by using 6 web based programs.

Gene	Chromosome: position	SNP ID	Variations		1000G MAF	Predictions					
			DNA	Protein		Polyphen	Vario watch	Mutation taster	SIFT	Mutation Assessor	LRT
ZNF860	3: 32,032,397	rs4955216	c.1826G>T	p.R609L	0.4307	Benign	Low	Polymorphism	N/A	Neutral	N/A
UBP1	3: 33,458,266	rs3736563	c.326A>G	p.N109S	0.4790	Benign	**High**	Polymorphism	Tolerated	Neutral	Neutral
OXSR1	3: 38,271,881	rs6599079	c.911C>T	p.T304I	0.1893	Benign	Low	Polymorphism	Tolerated	Neutral	Neutral
SLC22A14	3: 38,347,633	rs34043027	c.116G>A	p.R39H	0.0751	Benign	Low	Polymorphism	Tolerated	Low	Neutral
SLC22A14	3: 38,347,851	rs2073714	c.334A>G	p.K112E	0.0751	Benign	**High**	Polymorphism	Tolerated	Neutral	Neutral
SLC22A14	3: 38,355,225	rs73064822	c.1171G>A	p.V391I	0.1669	Benign	**High**	Polymorphism	Tolerated	Low	Neutral
SLC22A14	3: 38,357,817	rs2070492	c.1535C>T	p.A512V	0.1068	**Possibly damaging**	**High**	Polymorphism	Tolerated	Low	Neutral
SCN10A	3: 38,739,285	rs561166361	c.5426A>G	p.K1809R	0.0002	**Probably damaging**	**High**	**Disease causing**	**Damaging***	**Medium**	**Deleterious**
SCN10A	3: 38,768,300	rs57326399	c.2884A>G	p.I962V	0.2107	Benign	Low	Polymorphism	Tolerated	Neutral	Neutral
SCN10A	3: 38,768,457	rs567269429	c.2727C>A	p.N909K	0.0002	Benign	**High**	**Disease causing**	Tolerated	**Medium**	Neutral
SCN11A	3: 38,888,735	rs72869687	c.4826C>T	p.T1609I	0.0693	Benign	**High**	Polymorphism	Tolerated	Neutral	Neutral
XIRP1	3: 39,225,752	rs61736128	c.5185G>C	p.V1729L	0.1030	Benign	Low	Polymorphism	Tolerated	Neutral	Unknown
XIRP1	3: 39,226,436	rs58805228	c.4501G>A	p.V1501M	0.1030	**Possibly damaging**	**High**	Polymorphism	**Damaging**	**Medium**	Unknown
TTYH2	17: 72,233,549	rs371502920	c.531G>A	p.M177I	0.0008	**Possibly damaging**	**High**	**Disease causing**	Tolerated	**Medium**	**Deleterious**
LLGL2	17: 73,567,846	rs1661715	c.2275C>T	p.P759S	0.3099	**Possibly damaging**	Low	Polymorphism	Tolerated	Low	**Deleterious**
LLGL2	17: 73,570,718	rs1126939	c.2981C>G	p.P994R	0.3153	Benign	Low	Polymorphism	**Damaging***	N/A	N/A
MGAT5B	17: 74,868,836	rs732186	c.5A>G	p.H2R	0.0094	Benign	**High**	Polymorphism	N/A	Neutral	N/A
TMC6	17: 76,121,031	rs34712518	c.572G>A	p.G191D	0.1038	Benign	Low	Polymorphism	Tolerated	Low	Neutral
DNAH17	17: 76,503,593	rs9896398	c.4531A>G	p.T1511A	0.4215	Benign	Low	Polymorphism	Tolerated	Neutral	N/A

* Low confidence

Table S3. Genotyping and segregation analyses of selected four variations on *SCN10A*, *XIRP1*, and *TTYH2* in the UBRS082 family and in normal control subjects.

Gene	Variation	Family (UBRS082)		Control subjects (N=180)	
		Segregation	LOD	No. of detected samples	Alleles frequency
SCN10A	N909K	Yes	3.31	0	0
SCN10A	K1809R	Yes	3.31	0	0
XIRP1	V1501M	Yes	3.31	24	0.067
TTYH2	M177I	Yes	3.31	3	0.008

Table S4. A summary of *SCN10A* variations identified in additional 180 patients with KSD.

No. of variations	SNP ID	Location	Nucleotide change	Amino acid change	Amino acid group change	No. of detected samples (n=180)	MAF
1	rs190176472	Exon 1	c.53C>T	P18L	Yes	1	0.0028
2	rs368338265	Exon 1	c.144A>C	E48D	No	2	0.0056
3	rs144270136	Exon 1	c.268C>T	R90W	Yes	2	0.0056
4	rs142884499	Exon 2	c.365C>T	T122M	Yes	1	0.0028
5	rs370917734	Intron 2	c.390-9G>A	-	-	2	0.0056
6	rs73826327	Intron 4	c.600-43G>C	-	-	39	0.1250
7	rs74717885	Exon 5	c.618A>G	I206M	No	39	0.1250
8	Novel	Intron 5	c.692-9T>G	-	-	1	0.0028
9	rs7641844	Intron 6	c.884-13T>C	-	-	108	0.3000
10	rs62244070	Exon 9	c.1284G>A	E428E	No	58	0.1833
11	rs7617919	Exon 11	c.1476C>T	L492L	No	3	0.0083
12	rs544900253	Intron 13	c.2106-45G>A	-	-	1	0.0028
13	rs9830687	Intron 14	c.2280+35C>T	-	-	28	0.0778
14	rs7374804	Exon 16	c.2850A>G	K950K	No	23	0.0638
15	rs57326399	Exon 16	c.2884A>G	I962V	No	113	0.4250
16	rs59468016	Exon 16	c.2937C>T	G979G	No	85	0.2361
17	rs11129804	Intron 16	c.3088-18T>C	-	-	80	0.2222
18	rs6795970	Exon 17	c.3218T>C	V1073A	Yes	88	0.2444
19	rs12632942	Exon 18	c.3275T>C	L1092P	No	5	0.0167
20	rs6771157	Exon 19	c.3393C>G	T1131T	No	135	0.4667
21	rs560631745	Exon 19	c.3445G>A	V1149M	No	1	0.0028
22	rs758184076	Exon 20	c.3597C>T	F1199F	No	14	0.0389
23	rs6781740	Intron 21	c.3805-41G>A	-	-	77	0.2139
24	rs6790627	Exon 25	c.4323A>G	K1441K	No	56	0.1556
25	rs191624001	Exon 26	c.4515G>A	T1505T	No	6	0.0167
26	rs78425180	Exon 27	c.4710G>A	T1570T	No	10	0.0278
27	rs6599242	Exon 27	c.4866C>T*****	S1622S	No	17	0.0472
28	rs146999807	Exon 27	c.5047C>T	P1683S	No	3	0.0083
29	rs142813340	3’ UTR	c.*40T>A	-	-	12	0.0333

*****: T is reported as major allele in SNP database, n: number of patients with nephrolithiasis, MAF: Minor allele frequency.

Table S5. Prediction of impacts of amino acid changes on the protein structure and function of $Na_V1.8$ α subunit resulting from the identified variations in exons of *SCN10A* by using 6 web-based programs.

SNP ID	Location	Nucleotide change	Amino acid change	1000G MAF	Function Prediction					
					Polyphen2	Vario Watch	MutationTaster	SIFT	MutationAssessor	LRT
rs190176472	Exon 1	c.53C>T	P18L	0.0006	**Probably damaging**	Medium	**Disease causing**	Tolerated	Low	Neutral
rs368338265	Exon 1	c.144A>C	E48D	N/A	Benign	Low	Polymorphism	Tolerated	Low	Neutral
rs144270136	Exon 1	c.268C>T	R90W	0.0054	**Probably damaging**	**High**	**Disease causing**	**Damaging**	**Medium**	Neutral
rs142884499	Exon 2	c.365C>T	T122M	0.0004	Benign	**High**	Polymorphism	**Damaging**	**Medium**	Neutral
rs74717885	Exon 5	c.618A>G	I206M	0.0419	Benign	Low	Polymorphism	Tolerated	Neutral	Neutral
rs62244070	Exon 9	c.1284G>A	E428E	0.2075	Benign	Low	Polymorphism	Tolerated	N/A	N/A
rs7617919	Exon 11	c.1476C>T	L492L	0.2077	Benign	Low	Polymorphism	Tolerated	N/A	N/A
rs7374804	Exon 16	c.2850A>G	K950K	0.1360	Benign	Low	Polymorphism	Tolerated	N/A	N/A
rs57326399	Exon 16	c.2884A>G	I962V	0.2107	Benign	Low	Polymorphism	Tolerated	Neutral	Neutral
rs59468016	Exon 16	c.2937C>T	G979G	0.1905	Benign	Low	Polymorphism	Tolerated	N/A	N/A
rs6795970	Exon 17	c.3218T>C	V1073A	0.2420	Benign	Low	Polymorphism	Tolerated	Neutral	Neutral
rs12632942	Exon 18	c.3275T>C	L1092P	0.2196	Benign	Low	Polymorphism	Tolerated	N/A	N/A
rs6771157	Exon 19	c.3393C>G	T1131T	0.2196	Benign	Low	Polymorphism	Tolerated	N/A	N/A
rs560631745	Exon 19	c.3445G>A	V1149M	0.0002	**Probably damaging**	**High**	**Disease causing**	**Damaging**	**Medium**	**Deleterious**
rs758184076	Exon 20	c.3597C>T	F1199F	N/A	Benign	Low	Polymorphism	Tolerated	N/A	N/A
rs6790627	Exon 25	c.4323A>G	K1441K	0.2424	Benign	Low	Polymorphism	Tolerated	N/A	N/A
rs191624001	Exon 26	c.4515G>A	T1505T	0.0030	Benign	Low	Polymorphism	Tolerated	N/A	N/A
rs78425180	Exon 27	c.4710G>A	T1570T	0.0579	Benign	Low	Polymorphism	Tolerated	N/A	N/A
rs6599242	Exon 27	c.4866C>T	S1622S	0.0753	Benign	Low	Polymorphism	Tolerated	N/A	N/A
rs146999807	Exon 27	c.5047C>T	P1683S	0.0024	Benign	Low	Polymorphism	Tolerated	Low	Neutral

Table S6. Some clinical and laboratory data of the members of the UBRS094 family.

Sample No.	Gender	Age	Age of Onset*	KUB result	Site/Side of stone	No. of stone	Treatment	Urine pH	Other Symptoms				Diagnosis
									Dysuria	Hematuria	Pass Stone	Turbid Urine	
II:1	Male	56	40	Negative	N/A	N/A	No	5.3	Yes	Yes	Yes	Yes	renal stone (strong clinical history[1])
II:2	Female	34	24	Positive	Renal/ Both	4	Surgery	6.7	Yes	Yes	No	Yes	renal stone (KUB[2])
II:3	Female	33	30	Positive	Renal/Left	1	No	5.8	Yes	Yes	No	No	renal stone (KUB)
III:1	Female	26	25	Positive	Renal/Left	1	No	5.9	Yes	No	No	Yes	renal stone (KUB)
II:4	Female	56	-	Negative	No	-	No	5.0	No	No	No	No	no stone

[1] The patient had strong clinical history as justified from the presence of several symptoms associated with kidney stone, especially hematuria and stone passage.

[2] The patient had positive results of kidney–ureter–bladder (KUB) radiography.

* Age at the onset of kidney stone disease.

Table S7. Average time constant of inactivation at 0 mV and persistent currents associated to wild-type and mutant $Na_V1.8$ channels.

$Na_V1.8$	Time constant of inactivation (0 mV)		Persistent currents (0 mV)	
	ms	n	% of I peak	n
Wild-type	4.83 ± 0.79	23	8.62 ± 1.04	23
I1706V	2.99 ± 0.45	12	2.24 ± 1.16*	12
N909K	4.68 ± 0.59	22	3.61 ± 1.50*	22
K1809R	7.49 ± 0.72**	24	8.45 ± 1.82	23
N909K/ K1809R	6.12 ± 0.89	24	10.72 ± 1.62	24

Time constants were obtained by fitting decaying currents with a single-exponential function. Persistent currents were the mean current between 93-98 ms. *$p < 0.05$, **$p < 0.01$ vs wild-type (unpaired t-test).

Table S8. Average recovery time constant (at -80 mV) for fast and slow inactivation of wild-type and mutant $Na_V1.8$ channels.

$Na_V1.8$	Recovery time constant (ms)		n
	Fast component	Slow component	
Wild-type	3.48 ± 0.36	82.35 ± 25.62	11
I1706V	4.84 ± 1.02	110.2 ± 30.61	10
N909K	4.00 ± 0.73	63.91 ± 10.01	16
K1809R	3.71 ± 0.50	70.38 ± 16.15	16
N909K/ K1809R	3.82 ± 0.49	89.67 ± 22.82	11

No significant difference was found between wild-type and mutant $Na_V1.8$ channels.

Table S9. Some characteristics of the patients with kidney stone disease (KSD) and normal control subjects.

Characteristics	Patients (n)	%	Controls (n)	%
**Gender**				
Female	77	42.78	81	45.00
Male	103	57.22	99	55.00
**Age**				
Mean (years)	44.1 ± 12.8	-	44.3 ± 12.5	-
Range (years)	22-73	-	21-75	-
**Familial history**				
Yes	128	71.11	-	-
No	52	28.89	-	-
**Stone number**				
Single	50	27.78	-	-
Multiple	120	66.67	-	-
Unknown	10	5.55	-	-
**Position of stone**				
Kidney	163	90.55	-	-
Ureter	5	2.78	-	-
Kidney and ureter	12	6.67	-	-

Table S10. PCR primers for genotyping 4 genetic variations in *SCN10A*, *SCN10A*, *XIRP1*, and *TTYH2* genes in the members of UBRS082 family and 180 normal control subjects.

Gene	Variation	Nucleotide sequence (5'→3')	Number of nucleotides	Tm (°C)	Product size (bp)
SCN10A	N909K	TGGGGCTCAGCTTAGAGATTGA	22	63.5	
		ATCCGTGCCAGGGCCACCTGCCG	23	76.9	162
SCN10A	K1809R	CCAAAACCCAATCGAAATATAC	22	57.5	
		AGAGTGGTTGCTATTGGTTCAT	22	57.8	206
XIRP1	V1501M	GCCCTGCGGAGGCTCTTTGTGGCC	24	77.6	
		TTGCCAAGGTCTGTTCCTTCAG	22	63.8	160
TTYH2	M177I	AAGGTGGACCTAGAGCAGCA	20	60.0	
		GAGAGCTGAACAACAACGC	19	57.1	119

Table S11. PCR primers for amplifications of all exons of *SCN10A* for screening and sequencing of its genetics variations.

Fragments No.	Region	Primer name	Nucleotide sequence (5'→3')	Number of nucleotides	Tm (°C)	Product size (bp)
F1	Exon 1	F1F	CAGTGGGCAAGCTGTCAC	18	58.9	436
		F1R	AGGGCCAAGGAGGACTTTCT	20	61.4	
F2	Exon 2	F2F	CCTGAGATAATGCCTCTCATGT	22	58.3	275
		F2R	GTACTGGACACAGTAGGCAAGG	22	58.8	
F3	Exon 3	F3F	GTAATCATTCAGCATCAAGGTG	22	57.2	240
		F3R	AAGACAAAGACTGCCAAGTGAA	22	59.0	
F4	Exon 4	F4F	GGTCATCCCTCCCTCCTG	18	60.4	283
		F4R	CCCTGTCTCCTACCCACCA	19	60.9	
F5	Exon 5	F5F	CCCAGGTTATCCTTATTTATGCT	23	57.7	265
		F5R	TCTTTGCCCTGGAACCTTAC	20	59.1	
F6	Exon 6	F6F	AGAGATGAAGGAGGATGGCTA	21	57.9	365
		F6R	ACACAGGGGCTCCATATCC	19	59.7	
F7	Exon 7	F7F	TCACTTTGGAAGACTTGGGAAT	22	59.9	240
		F7R	GCCCATAATATGCCAAGGAC	20	59.2	
F8	Exon 8	F8F	AAGTGTCCAAGATCATCCTGCT	22	60.1	364
		F8R	CTCCTCCAGGAAAAGCACAG	20	59.9	
F9	Exon 9	F9F	TCCTGGAGGAGGCTGACTT	19	59.3	367
		F9R	ACGGTGCCCTAATTGAAGTG	20	60.5	
F10	Exon 10	F10F	CATGTCTGGGGGACACATTC	20	61.2	322
		F10R	CCTCTTCCCACTCCAACTCA	20	60.2	
F11	Exon 11	F11F	AGAGGGAAGTGTTGGGCTCT	20	60.5	449
		F11R	TTGGTGATACTCAGGCTTCTTG	22	61.3	
F12	Exon 12	F12F	TGGCCAATAAGAGGCTGAAT	20	59.6	266
		F12R	TTGCTTCTGCTGCTCACATG	20	61.3	
F13	Exon 13	F13F	AGAGGATGACCGCAGAATTG	20	60.9	385
		F13R	GAACTGCACCCTGCCATC	18	58.9	
F14	Exon 14	F14F	CCTCAGGAGCCCACAGATAA	20	60.2	387
		F14R	CCCAGAGCACAGTGTCTTCC	20	60.8	

Table S11. (Continued).

Fragments No.	Region	Primer name	Nucleotide sequence (5’→3’)	Number of nucleotides	Tm (°C)	Product size (bp)
F15/1	Exon 15	F15/1F	TCTTTGGTTTCCAGGCAAAA	20	60.6	310
		F15/1R	TGGAAGAAGTCGTGCATGTG	20	60.9	
F15/2	Exon 15	F15/2F	GGGAAAACTACCGTAACAACC	21	57.3	309
		F15/2R	TCTGGGCATAAAAATGCAGA	20	59.2	
F16/1	Exon 16	F16/1F	CTAACCCGGTAGGCCAATGT	20	61.1	326
		F16/1R	GTTGGCAGCAATGTGGTTC	19	60.1	
F16/2	Exon 16	F16/2F	CCCACTCTCCAGCTCCAAG	19	60.9	323
		F16/2R	TCCTCTGCATTTCCCTTTTG	20	60.1	
F17	Exon 17	F17F	TGATGTGCAAGATCCCTTCA	20	60.2	298
		F17R	ACCCTCCAGCCTCTACCAG	19	59.2	
F18	Exon 18	F18F	CAAACATGGGTGCAGTTGAA	20	60.5	300
		F18R	TGGATCCCAGGTGAGTGTTC	20	60.9	
F19	Exon 19	F19F	TGGGTTTCCTAGCAGAATGG	20	60.1	339
		F19R	AGAACCCACTGATGCAGCTC	20	60.4	
F20	Exon 20	F20F	GCTGGGTCCTTCCCTATCAG	20	60.9	341
		F20R	GGTCTCTGAGCTTCCTTTGC	20	59.1	
F21	Exon 21	F21F	GGGAGTTCCTGTCTCCTTCC	20	60.1	240
		F21R	GTGCCTGGCCAGATGAGA	18	60.9	
F22/1	Exon 22	F22/1F	CTCATGAAGCAGCCTGAATG	20	59.5	311
		F22/1R	CTGCCAGTGGAGTTTTGAAT	20	57.8	
F22/2	Exon 22	F22/2F	TGTACCTTTGTCGATTGTGAAT	22	57.1	225
		F22/2R	GCAAAGTCCCCACATAGCAT	20	59.9	
F23	Exon 23	F23F	CTCTCACTGGGCCACCAT	18	59.6	230
		F23R	ACCAGGGTCATCGTCACTTC	20	59.9	
F24	Exon 24	F24F	TGTCTTAGGCTTGGCTTTCA	20	58.6	290
		F24R	AGAGCTGGGCAGGGAACA	18	61.9	
F25	Exon 25	F25F	CCTTTCTCTCCTGCCTGTTC	20	59.0	248
		F25R	AGGCATAGACTGTCATGTTGGA	22	59.6	

Table S11. (Continued).

Fragments No.	Region	Primer name	Nucleotide sequence (5'→3')	Number of nucleotides	Tm (°C)	Product size (bp)
F26/1	Exon 26	F26/1F	TGTGGAGTGTGATGGAGACAA	21	60.1	285
		F26/1R	GACACATTCGCCTGTGAAGA	20	59.8	
F26/2	Exon 26	F26/2F	AGACGAAAATTCTGGGCAAA	20	59.6	236
		F26/2R	TTGGTTGGTTATTTCCTTGG	20	57.4	
F27/1	Exon 27	F27/1F	CCAAGCAAAGCAGAGTGTCA	20	60.1	269
		F27/1R	AACAGCCCGATGTTGAAGAG	20	60.2	
F27/2	Exon 27	F27/2F	ATCCGCACACTGCTCTTTG	19	60.0	272
		F27/2R	GGCAGATTGGGGTCACAGTA	20	60.9	
F27/3	Exon 27	F27/3F	AGCCCCATCCTCAACACA	18	60.1	297
		F27/3R	GTCCGAGAGAGCAGAAAAGG	20	59.1	
F27/4	Exon 27	F27/4F	CCCTGAGTGAGGACGACTTT	20	59.3	334
		F27/4R	AGAGTGGTTGCTATTGGTTCA	21	57.4	
F27/5	Exon 27	F27/5F	TGGAGGAGAAGTTTATGGCAAC	22	60.5	293
		F27/5R	CTCATAGGACGGTGGGAATG	20	60.3	
F27/6	Exon 27	F27/6F	CCCAGACAAATCTGAAACTGC	21	59.7	247
		F27/6R	TGTAGCTGGGTGTGATCTGC	20	59.9	

Table S12. PCR primers for analysis of *SCN10A* expression.

Gene	Nucleotide sequence (5'→3')	Number of nucleotides	Tm (°C)	Product size (bp)
SCN10A exon 2-3	CATTTATGGTGCTGAACAAAGG	22	52.7	
	TTTCTCTGGAAGGTCAGTTCG	21	54.2	194
ACTB	GCTCGTCGTCGACAACGGCTC	21	62.3	
	CAAACATGATCTGGGTCATCTTCTC	25	55.6	353

Supplementary methods

Genome-wide linkage analysis

Genome-wide linkage analysis was performed in a selected family (UBRS082) with a maximal ELOD of 3.31, containing 17 members (7 affected and 10 unaffected), by using either SNP 10K array (Human Mapping 10K 2.0; Affymetrix, USA) or SNP 1M array (Genome-Wide Human SNP Array 6.0; Affymetrix, USA) for SNP genotyping of the DNA samples from all 17 members.

The experimental steps for SNP genotyping by SNP 10K array (Human Mapping 10K 2.0) were conducted by following the manufacturer's instruction. To obtain higher genomic coverage, SNP genotyping was repeated by using SNP 1M array (Genome-Wide Human SNP Array 6.0). The DNA samples from all 17 members were sent to the Functional Genomics Shared Resource (Vanderbilt University, Tennessee, USA) for SNP genotyping. The SNP genotyping data from both arrays were analyzed by easyLINKAGE software[1] compiling many useful types of software for linkage analysis including FastLink, SuperLink, SPLink, GeneHunter, Merlin, Simwalk2, PedCheck and FastSLink. The analyses were carried out by both parametric and non-parametric modes, and in both two-point and multi-point methods. Regions with significant (LOD >2.80) or highest LOD scores were selected for further investigations.

Exome sequencing

Two DNA samples of affected members (II:5 and II:6) and one unaffected member (II:7) were taken for exome capturing by IlluminaTruSeqExome Enrichment kits (Illumina,California, USA) and sequencing by IlluminaHiSeq 2000 (Illumina,California, USA). The DNA samples were sent to the Axeq Asia (Seoul, South Korea), for the exome capturing and sequencing steps by following the Illumina protocols. The reads were mapped against UCSC hg19 by Burrows-Wheeler Aligner and variations (SNPs and Indels) and were detected by Sequence Alignment/Map.

Genetic variations acquired from exome sequencing data in the chromosomal regions with high LOD scores (>2.80) were taken for the analysis. The variations outside exonic regions were initially excluded as they were likely to be non-disease causing polymorphisms. Since KSD in the UBRS082 family was inherited as autosomal dominant mode, the genetic variations that were shared in the two affected family members but not observed in the unaffected family member were selected for further analyses. The variations in exons that caused non-synonymous changes, stop gain/loss variants or short insertions or deletions (indels) were considered for further analysis.

The possible impacts of amino acid changes on structure and function of protein were predicted by using 6 web-based programs: Polymorphism Phenotyping v2 (PolyPhen-2),[2] VarioWatch,[3] MutationTaster,[4] Sorting Intolerant From Tolerant (SIFT),[5] MutationAssessor,[6] and Likelihood Ratio Test (LRT).[7] The impact of exon-intron boundaries on mRNA splicing process was evaluated using ESEfinder 2.0.[8] Multiple amino acid sequence alignment of $Na_V1.8$ α subunit of voltage-gated sodium channel from human, chimpanzee, orangutans, gibbon, dog, cow, mouse, rat, anole and chicken were carried out by using ClustalW2 program.

Genotyping of genetic variations in family members and normal control subjects

Nucleotide sequences of the genes of interest were acquired from the GenBank database for designing polymerase chain reaction (PCR) primers (Table S10). The specific primer-pairs generated amplicons with the sizes between 119 and 206 bp, covering genetic variations of interest for genotyping DNA samples of family members and normal control subjects by using PCR-high resolution melting (PCR-HRM) analysis[9,10] and either PCR-restriction fragment length polymorphism (PCR-RFLP) or derived cleaved amplified polymorphic sequences (dCAPS) method.[11] The genetic variations that were observed by these genotyping methods in all DNA samples were confirmed by Sanger DNA sequencing.

Screening of genetic variations in *SCN10A* by PCR-HRM method

The whole nucleotide sequence of *SCN10A* was obtained from the GenBank database. Thirty-six pairs of PCR primers covering 27 exons and their exon-intron boundaries were designed (Table S11) and synthesized. Genetic variations in *SCN10A* in DNA samples of the patients with KSD were screened by PCR-HRM method by using a LightCycler 480 II machine (Roche Diagnostics International Ltd., Rotkreuz, Switzerland). The PCR mixture contained Resolight dye (Roche Diagnostics GmbH, Mannheim, Germany) for detection of PCR products and for analysis of their melting curves. The melting curve was normalized for temperature-shifted difference plot by Gene Scanning 1.5.0 software (Roche Diagnostics International Ltd., Rotkreuz, Switzerland). The PCR products with melting profile different from the normal pattern were further analyzed by Sanger DNA sequencing.

DNA sequencing

PCR product for DNA sequencing was prepared in PCR reaction volume of 50 µL containing 200 ng genomic DNA in 1x reaction buffer, 0.5 µM of each primer, 0.2 mM dNTP, 2.0 mM $MgCl_2$ and 1.25 unit of DNA polymerase (HS Prime *Taq* DNA polymerase, GENET BIO Inc., Daejeon, South Korea) by using a TProfessional standard thermocycler (Biometra GmbH, Goettingen, Germany). The PCR product was purified before processing for direct DNA sequencing by the Sanger method. The sequencing reaction was carried out by using BigDye™ Terminator, which was conducted by a service provider - First BASE Laboratories Sdn Bhd (Selangor, Malaysia). The sequencing data was analyzed by comparing with a reference nucleotide sequence by multiple sequence alignment using ClustalW2 program.

Human kidney tissues

The study using human kidney tissues was approved by the Human Research Ethics Committee, Siriraj Institutional Review Board, Mahidol University. Human fresh frozen tissues from the patients without KSD were obtained from remaining biopsy specimens from the Department of Pathology, Faculty of

Medicine Siriraj Hospital, Mahidol University. These tissues were used for the studies of *SCN10A* mRNA expression by reverse transcription and polymerase chain reaction (RT-PCR) method, and its encoded protein ($Na_V1.8$ α subunit of voltage-gated sodium channel) expression by Western-blot analysis, immunohistochemistry and double immunofluorescence staining.

Reverse transcription and polymerase chain reaction (RT-PCR)

Total RNAs were extracted from human fresh frozen kidney tissues, human sperm, and HEK293T cells by Trizol reagent (Invitrogen, Carlsbad, CA, USA), following the manufacturer's protocol. RNA was then reverse-transcribed into cDNA by using oligo dT and SuperScript^{TM} III First-Strand synthesis system (Invitrogen, Carlsbad, CA, USA). *SCN10A* cDNA was examined by PCR amplifications of a region covering exons 2-3 of *SCN10A*, and *ACTB* cDNA was amplified to serve as an internal control. The primer sequences are shown in Table S12. The RT-PCR products from kidney tissues were further analyzed by Sanger DNA sequencing.

Western-blot analysis

Proteins were extracted from human kidney tissue sections (20-μm thick) in 2x SDS buffer or from transfected cells in RIPA. Ten μl of human kidney tissue protein or 15 μl of protein from transfected cells of each sample was separated by SDS-polyacrylamide gel electrophoresis (PAGE). After electrophoresis, the proteins were transferred onto a nitrocellulose membrane by following a standard protocol. The membrane was blocked with 5% skim milk in TBST solution and incubated with rabbit anti-human $Na_V1.8$ α subunit of voltage-gated sodium channel (Abcam, Cambridge, UK) or mouse anti-SCN1B (Abcam, Cambridge, UK), followed by incubation with swine anti-rabbit antibody conjugated-horseradish peroxidase (HRP) (DakoCytomation, Glostrup, Denmark) or rabbit anti-mouse antibody conjugated-HRP (DakoCytomation, Glostrup, Denmark). Actin in each sample was detected as an internal control by using mouse anti-actin antibody (Sigma-Aldrich, Missouri, USA) and rabbit anti-mouse antibody conjugated-HRP (DakoCytomation, Glostrup, Denmark). Chemiluminescent signals generated by SuperSignal West Pico

Chemiluminescent Substrate (Thermo Scientific, Illinois, USA) were detected by using a G:BOXchemiluminescence imaging system (Syngene, Cambridge, UK).

Plasmid constructs, cell culture and transfections

The wild-type constructs, pcDNA5_Na$_V$1.8 and pcDNA5_Na$_V$1.8-venus, and reported gain-of-function mutation, pcDNA5_Na$_V$1.8_I1706V, were previously reported.[12,13] The wild-type constructs were used as templates to generate mutant constructs by PCR and site-directed mutagenesis method using *Pfx* DNA polymerase (Invitrogen, Carlsbad, CA, USA). Three additional mutant constructs, p.N909K, p.K1809R, and p.N909K/K1809R, of *SCN10A* were generated by the same method. This part of work was conducted at the Division of Molecular Medicine, Faculty of Medicine Siriraj Hospital, Mahidol University, Bangkok, Thailand. All mutant constructs were confirmed by Sanger DNA sequencing (First BASE Laboratories SdnBhd, Selangor, Malaysia).

HEK293 cells stably expressed SCN1B (HEK293-1B) generated for studying Na$_V$1.8 α subunit expression were cultured in Dulbecco's Modified Eagles Medium-F12 (DMEM-F12) supplemented with 10% (v/v) fetal bovine serum, 1.2% (v/v) pencillin-streptomycin solution (100 U/ml penicillin and 100 μg/ml streptomycin), and 200 μg/ml of G418 in a humidified CO_2 (5%) incubator at 37°C. The HEK293-1B cells were transfected with the wild-type or mutant plasmid constructs by using Lipofectamine™ 2000 (Invitrogen, Carlsbad, CA) according to manufacturer's instructions. The transfected cells were additionally incubated at 37°C, CO_2 for 48 hours prior to further investigations.

Immunohistochemistry staining

Human kidney tissues from remaining biopsy specimens were fixed in 4% paraformaldehyde and embedded in paraffin blocks. Then, 4-μm-thick tissues were serially cut from paraffin blocks and mounted onto glass slides coated with 1% (W/V) gelatin solution. After deparaffinization and rehydration, the kidney tissue sections were placed in sodium citrate buffer (10 mM sodium citrate, 2 mM EDTA, 0.05% tween-20,

pH 6.0) and subjected to heat retrieval at 70°C for 20 minutes. The sections were allowed to cool to room temperature and endogenous peroxidase activity was blocked by 3% hydrogen peroxide for 30 minutes. The sections were placed in 2% BSA for 30 minutes to block nonspecific bindings and incubated with a rabbit antibody against either Na_V 1.8 α subunit (1:20) (Abcam, Cambridge, UK), AQP1 (1:200) or AQP2 (1:50) in PBS/2% (w/v) BSA overnight. Normal rabbit IgG (Cell Signaling Technology, USA) in equal concentration to the primary antibody was used as isotype control. After washing, secondary antibody (swine) against rabbit antibody conjugated with horseradish peroxidase (HRP, Dako, Japan) at dilution 1:200 in PBS/2% BSA was applied and incubated for 6 hours. The sections were washed and the color was developed by incubating in 0.03% 3.3-diaminobenzidine tetrahydrochloride (DAB) for at least 2 minutes and counterstained with hematoxylin and eosin (H&E). The tissue sections were then mounted with xylene-based mounting medium (Leica Biosystems, Illinois, USA) and viewed under a Nikon Eclipse TE200 inverted fluorescent microscope (Nikon, Tokyo, Japan).

Double immunofluorescence staining

The human fresh-frozen kidney tissues were sliced into 2-4 μm thickness by a rotary microtome. The tissue section was placed on a glass slide and fixed with acetone for 10 minutes before incubation with rabbit anti-human Na_V1.8 α subunit of voltage-gated sodium channel (Alomone, Jerusalem, Israel) and mouse anti-alpha 1 Na^+/K^+ATPase (Abcam, Cambridge, UK) as the primary antibodies, followed by incubation with donkey anti-rabbit IgG conjugated with Alexa 488 fluorescein and goat anti-mouse IgG conjugated with Cy3 fluorescein (Invitrogen, California, USA) as the secondary antibodies. The tissue sections were then incubated with PBS containing Hoechst 33258 (Molecular Probes), washed with PBS, and mounted with Fluorosave (DakoCytomation, Glostrup, Denmark). The stained tissue section was examined under a Olympus BX51 fluorescence microscope (Olympus, Tokyo, Japan).

Quantitative western-blot analysis

For quantitative Western-blot analysis, the transfected HEK293-1B cells were lysed with 100 µl of homogenization buffer [containing 150 mM NaCl, 1% Triton X-100, 20 mM Tris HCl (pH 7.5), 1 mM EDTA, 1 mM DTT, and 1x complete mini protease inhibitor (Sigma-Aldrich)], incubated and rotated at 4°C at least 30 minutes. Samples were centrifuged at 15,000 g for 20 minutes at 4°C, and the supernatant fraction was collected as total protein extraction. After quantification of the protein concentration by the Bradford assay (Bio-Rad), 0.5, 1.0 and 1.5 ug of proteins were separated by 4-12% SDS-PAGE, followed by electrophoretic transfer onto a nitrocellulose membrane and immunoblot analysis with the mouse anti-Pan Sodium Channel antibody and rabbit anti-actin antibody (Sigma-Aldrich, Missouri, USA) at 1:1,000 dilution.

Electrophysiological study

Transfected cells, which had been in 200 µg/ml G418 and 1 mM lidocaine for 24 hours (48 hours after transfection), were plated onto poly-L-lysine-coated cover slips ($1x10^5$ cells per 35-mm Petri dish; lidocaine was still present), and left overnight (10-12 hours). Then, they were exposed to culture media without lidocaine for at least three hours before subjected to an electrophysiological experiment. Whole-cell patch-clamp recording was conducted at room temperature (25-26°C), using an Axopatch 200B patch-clamp amplifier (Axon Instruments, Foster City, CA, USA) and a Digidata 1440A analog-to-digital converter (Axon Instruments). The pipette solution contained 120 mM CsF, 15 mM NaCl, 10 mM HEPES, and 10 mM EGTA (pH 7.25 with CsOH). The extracellular bath solution contained 140 mM NaCl, 4.7 mM KCl, 1 mM $CaCl_2$, 1.3 mM $MgCl_2$, 5 mM HEPES, and 11 mM glucose (pH 7.4 with NaOH). To block endogenous tetrodotoxin (TTX)-sensitive Na^+ currents, 0.3 µM TTX was always included in the bath solution to block tetrodotoxin-sensitive ($Na_V1.7$, $Na_V1.2$, $Na_V1.3$) and the tetrodotoxin-resistant $Na_V1.5$ which is expressed at low and variable levels in these cells[14] and would be 60% inactivated at -80 mV, according to Wang et al., 2015.[15] Pipette resistance was 3.50 ± 0.08 MΩ (mean ± SEM), yielding maximal voltage errors of -2.46 ±

0.10 mV. Pipette potential was adjusted to zero before seal formation; liquid junction potential was not corrected. Holding potential (V_h) was -80 mV.

Currents were measured after leak subtraction. Current–voltage (I–V) curves were constructed from peak currents in response to a series of 100-ms step depolarization, at 5 s intervals, from -80 mV V_h to potentials between -60 and +60 mV, in 10 mV increments. Current density was calculated by normalizing peak currents with cell capacitance. Conductance (G) at each voltage (V) was calculated using the equation G = I / ($V-V_{rev}$) (I, peak current; V_{rev}, reversal potential). Individual conductance-voltage curve was plotted and fitted with a Boltzmann equation:

$$G = Gmin + \frac{Gmax - Gmin}{1 - exp\,\frac{V_{1/2} - V}{k}}$$

where conductance (G) is a function of the membrane potential (V); $V_{1/2}$ is the half maximal activation voltage; and k is the slope factor. Average $V_{1/2}$ and k of each group was used to construct the group's conductance-voltage curve.

Steady-state fast inactivation was assessed with a series of 500-ms prepulses (-90 to +10 mV in 5-mV increments) followed by a 40-ms test pulse to 0 mV to assess the available channels. Slow inactivation was assessed with 30-s prepulses at potentials ranging from -110 to +20 mV, followed by a 30-ms hyperpolarization at -80 mV to allow channel recovery from fast inactivation, and a 50-ms test pulse to 0 mV. Peak inward currents obtained during test pulses of steady-state fast-inactivation and slow-inactivation protocols were normalized to maximal peak current and fitted with the Boltzmann equation as a function of the inactivating voltage. Steady-state inactivation curves were constructed from average $V_{1/2}$ and k of each group.

The time constant of inactivation was obtained by fitting decaying currents with the standard exponential equation:

$$I = A * \exp(-t/\tau) + C$$

where A is the initial current amplitude, t is time, τ is the time constant of decay, and C is the asymptotic minimum to which the currents decay. Persistent currents were measured as mean current amplitude between 93 and 98 ms after the onset of depolarization,[13] and are presented as percentage of the peak current.

Recovery of $Na_V1.8$ channels from fast inactivation was examined using a two-pulse protocol. A prepulse (100 ms, 0 mV) was followed by a test pulse (10 ms, 0 mV), with interpulse intervals varying from 2 to 600 ms (-80 mV). Recovery rates were measured by normalizing peak current during the test pulse to that during the prepulse. Recovery time constants were calculated using two-phase exponential association. All curve-fitting were done using GraphPad PRISM 5® (GraphPad Software, San Diego, CA, USA).

Statistics

Data sets were collected from at least three independent experiments. Values are expressed as mean ± SEM (standard error of the mean). Kolmogorov–Smirnov test was used to test the normality of all data sets. Either unpaired t-test or Mann–Whitney U test, as appropriate, was employed to compare the mean between two different groups. One-way analysis of variance (ANOVA) followed by the Sidak's multiple comparison test was used for statistically significant differences between the means of two or more independent groups. Differences with $p < 0.05$ were considered statistically significant.

References

1. Lindner, T.H. & Hoffmann, K. easyLINKAGE: a PERL script for easy and automated two-/multi-point linkage analyses. *Bioinformatics* **21**, 405-7 (2005).
2. Adzhubei, I.A. *et al.* A method and server for predicting damaging missense mutations. *Nat Methods* **7**, 248-9 (2010).
3. Cheng, Y.C. *et al.* VarioWatch: providing large-scale and comprehensive annotations on human genomic variants in the next generation sequencing era. *Nucleic Acids Res* **40**, W76-81 (2012).
4. Schwarz, J.M., Rodelsperger, C., Schuelke, M. & Seelow, D. MutationTaster evaluates disease-causing potential of sequence alterations. *Nat Methods* **7**, 575-6 (2010).
5. Kumar, P., Henikoff, S. & Ng, P.C. Predicting the effects of coding non-synonymous variants on protein function using the SIFT algorithm. *Nat Protoc* **4**, 1073-81 (2009).
6. Reva, B., Antipin, Y. & Sander, C. Predicting the functional impact of protein mutations: application to cancer genomics. *Nucleic Acids Res* **39**, e118 (2011).
7. Chun, S. & Fay, J.C. Identification of deleterious mutations within three human genomes. *Genome Res* **19**, 1553-61 (2009).
8. Cartegni, L., Wang, J., Zhu, Z., Zhang, M.Q. & Krainer, A.R. ESEfinder: A web resource to identify exonic splicing enhancers. *Nucleic Acids Res* **31**, 3568-71 (2003).
9. Nettuwakul, C., Sawasdee, N. & Yenchitsomanus, P.T. Rapid detection of solute carrier family 4, member 1 (SLC4A1) mutations and polymorphisms by high-resolution melting analysis. *Clin Biochem* **43**, 497-504 (2010).
10. Reed, G.H., Kent, J.O. & Wittwer, C.T. High-resolution DNA melting analysis for simple and efficient molecular diagnostics. *Pharmacogenomics* **8**, 597-608 (2007).
11. Neff, M.M., Neff, J.D., Chory, J. & Pepper, A.E. dCAPS, a simple technique for the genetic analysis of single nucleotide polymorphisms: experimental applications in Arabidopsis thaliana genetics. *Plant J* **14**, 387-92 (1998).

12. Faber, C.G. *et al.* Gain-of-function Nav1.8 mutations in painful neuropathy. *Proc Natl Acad Sci U S A* **109**, 19444-9 (2012).

13. Huang, J. *et al.* Small-fiber neuropathy Nav1.8 mutation shifts activation to hyperpolarized potentials and increases excitability of dorsal root ganglion neurons. *J Neurosci* **33**, 14087-97 (2013).

14. He, B. & Soderlund, D.M. Human embryonic kidney (HEK293) cells express endogenous voltage-gated sodium currents and Na v 1.7 sodium channels. *Neurosci Lett* **469**, 268-72 (2010).

15. Wang, Y., Mi, J., Lu, K., Lu, Y. & Wang, K. Comparison of Gating Properties and Use-Dependent Block of Nav1.5 and Nav1.7 Channels by Anti-Arrhythmics Mexiletine and Lidocaine. *PLoS One* **10**, e0128653 (2015).
